# Assembly and Functional Coordination of Two Families of Metabolic Organelles in *Salmonella*

**DOI:** 10.1111/1751-7915.70301

**Published:** 2026-02-03

**Authors:** Ping Chang, Mengru Yang, Yu Chen, Tianpei Li, Marie Held, Lu‐Ning Liu

**Affiliations:** ^1^ Institute of Systems, Molecular and Integrative Biology University of Liverpool Liverpool UK; ^2^ Centre for Cell Imaging University of Liverpool Liverpool UK; ^3^ College of Marine Life Sciences, and Frontiers Science Center for Deep Ocean Multispheres and Earth System Ocean University of China Qingdao China

**Keywords:** bacterial microcompartment, carboxysome, encapsulation, organelle, propanediol‐utilisation microcompartment, protein assembly

## Abstract

Bacterial microcompartments (BMCs) are protein‐based organelles that spatially organise metabolic pathways in prokaryotes, playing critical roles in enhancing metabolic processes and microbe fitness. Notably, many bacterial species possess multiple types of BMCs. While recent studies have advanced our knowledge about the assembly and function of individual BMC types, the mechanisms governing the coexistence and interplay of distinct BMC families within a single bacterial cell remain poorly understood. Here, we engineered *Salmonella enterica* serovar Typhimurium LT2 to co‐express native 1,2‐propanediol utilisation (Pdu) BMCs and synthetic α‐carboxysomes (α‐CBs), providing a unique platform for dissecting their assembly dynamics and functional crosstalk. By exploiting super‐resolution fluorescence imaging, electron microscopy, biochemical and enzymatic assays, our studies demonstrate the formation of hybrid BMCs through the exchange of shell proteins between Pdu BMCs and α‐CBs, whereas cargo proteins exhibit only limited compatibility, highlighting the specificity of encapsulation mechanisms. Furthermore, the generated hybrid BMCs display altered mobility and enzymatic activities, revealing emergent properties arising from shell protein interchangeability. Our findings provide insights into the inherent structural plasticity and modular architecture of BMCs. More broadly, this study has implications for deciphering how bacterial cells modulate the construction and functions of diverse metabolic modules within a single cellular context and could inform the rational design and engineering of synthetic organelles and bio‐factories with tailored metabolic functions for biotechnological applications.

## Graphical abstract

Engineered *Salmonella enterica* co‐expressing native metabolosomes and synthetic carboxysomes formed hybrid bacterial microcompartments (BMCs) through shell protein exchange while maintaining cargo segregation, revealing the principles of BMC coexistence and modular assembly. Advanced understanding of in vivo BMC formation will inform the rational design of multifunctional organelles for metabolic engineering.

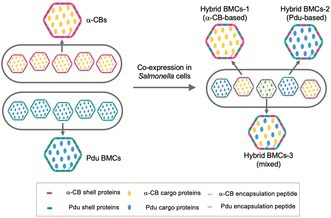

## 1 Introduction

Compartmentalization of metabolic pathways is fundamental for the regulation of cellular metabolism and energy flow in both eukaryotic and prokaryotic cells (Cornejo et al. 2014; Liu 2022). While eukaryotes employ membrane‐bound organelles to perform specific metabolic functions (Van Meer et al. 2008), many bacteria have evolved protein‐based organelles, known as bacterial microcompartments (BMCs), to sequester metabolic pathways from the cytosol (Axen et al. 2014; Liu et al. 2021). The BMC consists of a single‐layer proteinaceous shell that encapsulates both enzymatic cargo and substrates/metabolites to optimise metabolic processes (Bobik et al. 2015). To date, 68 types of BMC loci have been identified in 45 bacterial phyla (Sutter et al. 2021). These various BMC types play essential roles in diverse biological processes, including CO_2_ fixation, pathogenesis, and microbial ecology (Kerfeld et al. 2018; Liu 2021; Liu et al. 2021).

Based on their distinct functions, BMCs are classified into anabolic carboxysomes (CBs) and catabolic metabolosomes (Aussignargues et al. 2015; Liu 2021; Melnicki et al. 2021). CBs segregate ribulose‐1,5‐bisphosphate carboxylase/oxygenase (Rubisco) and carbonic anhydrase, and serve as CO_2_‐fixing organelles in all cyanobacteria and some chemoautotrophic and phototrophic bacteria (Kerfeld et al. 2018; Liu 2022). There are two distinct lineages of CBs, α‐carboxysomes (α‐CBs) and β‐carboxysomes (β‐CBs), which differ in the forms of encapsulated Rubisco and their associated protein composition (Shively et al. 1973; Liu 2022). Metabolosomes are responsible for the degradation of various carbon and nitrogen substrates (Brinsmade et al. 2005; Parsons et al. 2008; Stewart et al. 2020; Yang et al. 2025). A well‐known example is 1,2‐propanediol utilisation (Pdu) BMCs found in *Salmonella* and other enteric bacteria (Bobik et al. 1997, 1999; Prentice 2021), which provide a competitive growth advantage during gastrointestinal tract infections (Axen et al. 2014; Stewart et al. 2021).

Despite the functional and phylogenetic diversity, BMCs share common structural features. The BMC shell is constructed from a series of homologous proteins, organised predominantly into three forms: hexamers (BMC‐H, with one Pfam00936 domain), pseudohexameric trimers (BMC‐T, with two Pfam00936 domains) that tile the shell facets, and pentamers (BMC‐P, with one Pfam03319 domain) that cap the vertices of the polyhedral shell (Tsai et al. 2007; Cai et al. 2009; Klein et al. 2009). Conserved edge interfaces of shell proteins, along with specific encapsulation mechanisms, ensure proper and efficient assembly of the polyhedral shell and construction of intact BMCs (Trettel et al. 2024). Beyond the conserved architecture, BMCs exhibit inherent structural plasticity that enables them to modulate assembly and function in response to environmental variations (Liu et al. 2021), including morphological changes in size and shape, structural remodelling when specific components are missing, and regulation of shell permeability via vertex capping or dynamic protein–protein interactions (Sutter et al. 2016; Faulkner et al. 2017; Kodera et al. 2021; Huang et al. 2022; Sun et al. 2022).

The formation of structurally intact and fully functional BMCs within cells requires the coordinated spatial and temporal self‐assembly of thousands of individual BMC protein components. Notably, more than 20% of BMC‐containing bacterial species contain two or more classes of BMC gene operons, and some species encode up to six different types (Sutter et al. 2021). This suggests the potential for horizontal gene transfer of BMC loci among microbes (AbdulRahman 2013). Although the assembly and biogenesis of some BMCs have been characterised individually (Cameron et al. 2013; Yang et al. 2022, 2025), how bacteria simultaneously produce and maintain distinct types of BMCs within the same cell remains incompletely understood.

To address this question, we generated a framework to investigate the in vivo assembly and functional interplay of two distinct BMCs, native Pdu BMCs and heterologously engineered α‐CBs, within a single *Salmonella enterica* serovar Typhimurium ( *S. typhimurium* ) LT2 cell. *S. typhimurium* LT2 harbours the *pdu* operon in the native chromosome and expresses functional Pdu BMCs under endogenous regulatory control, thereby providing an ideal host for investigating the coordination of diverse BMC types. Using in vivo and in vitro approaches, we demonstrate the formation of hybrid BMCs in the cell, with the exchange of shell proteins from different origins. Hybrid BMCs exhibit altered protein composition and distinct enzymatic functions. Our findings provide insights into the assembly and modularity of BMCs within bacterial cells and offer important information required for the synthetic engineering of BMCs with novel properties to underpin biotechnological applications.

## 2 Materials and Methods

### 2.1 Generation of Constructs

The plasmids and oligonucleotides used in this study are listed in Tables S1 and S2, respectively. The Gibson Assembly strategy (NEBuilder HiFi DNA Assembly Master Mix, New England BioLabs, UK) was used for the fusion of interest genes, fluorescent tags, and promoters for the construction of plasmids. All backbones were linearized using PCR amplification. To construct the visualised α‐CB plasmids, mCherry was inserted into the C‐termini of CsoS1B and CbbL within the linearized pBAD33‐CBS1D vector (Sun et al. 2022). The *mcdAB* gene from the genome of *H. neapolitanus* (ATCC 23641 C2), with a His‐tag at the N‐terminus of McdA, was cloned into the pBAD vector to generate p*Hn*McdAB. sfGFP was tagged to the N‐terminus of McdA based on p*Hn*McdAB for visualisation of McdAB (p*Hn*McdAB‐1). To test the interchangeability of the individual components of α‐CBs, the genes encoding CsoS1A, CsoS1B, CsoS1C, CsoS1D, CsoS4A, CsoS4B, CsoS2, CbbLS (Rubisco), and CsoSCA were individually amplified from the genome of *H. neapolitanus* , and 3′ of each gene fused with *mCherry* was cloned into the linearized pBAD33 vector. The plasmids for the complementary experiment were generated by amplifying the nucleotide sequences of *pduN* from *S. typhimurium* LT2, *csoS4A* and *csoS4B* from *H. neapolitanus* , which were then cloned into the modified pBAD33 vector in which the P*ara* promoter was replaced by the P*tac* promoter. All constructs were transformed into *E. coli* DH5α cells and verified using colony PCR and DNA sequencing.

### 2.2 Bacterial Strains and Growth Conditions

The bacterial strains were derivatives of *S. enterica* serovar Typhimurium LT2. The rich medium used was LB‐Lennox medium (10 g·L^−1^ tryptone, 5 g·L^−1^ yeast extract, 5 g·L^−1^ sodium chloride), and the minimal medium used was no‐carbon‐E (NCE) medium (Vogel and Bonner 1956). The microcompartment‐inducing media (MIM) was NCE medium supplemented with 1 mM MgSO_4_, 0.6% 1,2‐PD, or 0.5% succinate (if applicable) as the sole carbon source and vitamin B_12_ (if applicable) (Yang et al. 2020, 2022). All medium components were purchased from Sigma‐Aldrich, except where specified. Antibiotics were added to the media as required at the following final concentrations: ampicillin, 100 μg·mL^−1^ kanamycin, 50 μg·mL^−1^; gentamicin, 20 μg·mL^−1^ in ddH_2_O; and chloramphenicol, 25 μg·mL^−1^ in ethanol.

### 2.3 Construction of Δ*parA* and Δ*minD* Mutant Strains

Δ*parA* and Δ*minD* were constructed using a scar‐less genome‐editing technique (Martinez‐Garcia and de Lorenzo 2011). Primers used in this study are listed in Table S2. 600–700 bp DNA products flanking regions of interest were amplified from *S. typhimurium* LT2 genomes, cloned into pEMG plasmid linearized by BamHI and EcoRI, and then transformed into *E. coli* S17‐1 λ*pir*. The donor strain, *E. coli* S17‐1λ*pir* with the pEMG derivative plasmid, was conjugated to *S. typhimurium* recipient strain. Transconjugants containing integrated pEMG were selected on M9 agar supplemented with 0.2% glucose and 50 μg·mL^−1^ kanamycin. The plasmid pSW‐2 from the LT2‐WT strain was cultured and mini‐prepped to increase the transformation efficiency due to *Salmonella*‐specific DNA modification of the plasmid. Transformants were selected on LB agar medium supplemented with 1 mM *m*‐toluate and 20 μg·mL^−1^ gentamicin. Kanamycin‐ and gentamicin‐sensitive colonies were screened by two passages in LB broth. *parA* is possibly an essential gene because Δ*parA* cannot be segregated from WT after two passages in LB. The pBAD33*tet*‐ParA plasmid for the complementary mutant experiment was generated by amplifying the nucleotide sequence of *parA* from *S. typhimurium* LT2 and was cloned into the modified pBAD33 vector in which the P*ara* promoter was replaced by the P*tetAR* promoter. The generated plasmid was transformed into the LT2‐WT strain as a recipient strain to construct Δ*parA*. Subsequent selection was supplemented with 25 μg mL^−1^ chloramphenicol and induced with anhydrotetracycline (1 mM). All the genomic mutant strains were verified using PCR and DNA sequencing.

### 2.4 Expression and Isolation of α‐Carboxysomes

Ten millilitres of *Salmonella* strains containing the pBAD33‐CBS1D vector were inoculated at 37°C in LB‐Lennox medium containing 25 μg·mL^−1^ chloramphenicol for overnight growth. The overnight LB culture was sub‐inoculated 1:100 in 800 mL MIM medium in 2 L flasks with 1 mM MgSO_4_, 0.5% succinate, and 25 μg·mL^−1^ chloramphenicol at 37°C. The expression of α‐CBs was induced by l‐arabinose (1 mM, final concentration) when the culture reached an OD_600_ of 0.4–0.6. Cells were grown at 18°C for 16 h with constant shaking at 120 rpm, and then harvested by centrifugation at 6000 ***g*** for 10 min. The purification of α‐CBs was performed as previously described (Li et al. 2020). Briefly, cell pellets were washed with TEMB buffer (10 mM Tris–HCl pH 8.0, 1 mM EDTA, 10 mM MgCl_2_, and 20 mM NaHCO_3_) and resuspended in the same buffer supplemented with 10% (v/v) cell lysis reagent (CelLytic B) and 1% protein inhibitor cocktail (PIC, 100X). Cells were lysed using a sonication regimen of 12 cycles (30 s on, 30 s off) at 125 W using a Q125 sonicator. Cell debris was removed by centrifugation at 12,000 ***g*** for 10 min, followed by centrifugation at 47,000 ***g*** to enrich α‐CBs. The pellets were resuspended in TEMB and loaded onto 10%–50% (w/v) sucrose gradients. After ultracentrifugation in a Beckman XL100K ultracentrifuge at 105,000 ***g*** for 30 min, the sucrose fraction was collected and stored at 4°C.

### 2.5 Expression and Isolation of Pdu BMCs

Overnight *S. typhimurium* LT2 culture was inoculated 1:100 in 400 mL MIM medium in 1 L flasks with 1 mM MgSO_4_, 0.6% 1,2‐PD, and 0.5% succinate. The cell culture was incubated at 37°C with shaking at 235 rpm until OD_600_ reached 1.0–1.2. Pdu BMCs were isolated using detergent treatment and differential centrifugation as previously described (Yang et al. 2020). Briefly, cells were harvested and washed twice with 40 mL of buffer A (50 mM Tris–HCl, 500 mM KCl, 12.5 mM MgCl_2_, and 1.5% 1,2‐PD, pH 8.0) and lysed using a 25 mL mixture of buffer A and BPER‐II (2:3, v/v, Thermo Fisher) supplemented with 5 mM 2‐mercaptoethanol, 1× PIC, 25 mg lysozyme, and 2 mg DNase I with 60 rpm shaking at room temperature for 50 min. Subsequently, Pdu BMCs were separated from cell debris by sequential centrifugation steps (12,000 ***g*** for 10 min to pellet cell debris, 20,000 ***g*** for 20 min to enrich Pdu BMCs). The BMC pellet was washed with 10 mL of a mixture of buffer A and BPER‐II (2:3, v/v) containing 1× PIC and subsequently resuspended in 0.2 mL of buffer B (50 mM Tris–HCl pH 8.0, 50 mM KCl, 5 mM MgCl_2_, 1% 1,2‐PD). To further remove cell debris, isolated Pdu BMCs were centrifuged at 12,000 ***g*** for 1 min, which was repeated thrice.

### 2.6 Expression and Isolation of Hybrid BMCs

Overnight *S. typhimurium* LT2 culture containing the pBAD33‐CBS1D vector and p*Hn*McdAB vector was sub‐inoculated 1:100 in 800 mL of MIM medium with 1 mM MgSO_4_, 0.6% 1,2‐PD, 150 nM vitamin B_12_, 25 μg·mL^−1^ chloramphenicol, and 100 μg·mL^−1^ ampicillin at 37°C. The expression of hybrid BMCs was induced by l‐arabinose (1 mM) when the culture reached an OD_600_ of 0.2–0.4. Cells were grown at 18°C for 24 h with shaking at 120 rpm, and then harvested by centrifugation at 6000 ***g*** for 10 min. Hybrid BMCs were isolated using the established purification protocols for α‐CB and Pdu BMCs, as mentioned above.

### 2.7 Carbon‐Fixation Assays

Rubisco activity was measured as previously described (Faulkner et al. 2017). Briefly, 5 μL of a 1 mg/mL purified sample in assay buffer (100 mM EPPS, pH 8.0; 20 mM MgCl_2_) was added to scintillation vials containing 25 mM NaH^14^CO_3_ and incubated at 30°C for 1 min. The reaction was initiated by adding ribulose 1,5‐bisphosphate (RuBP) to a final concentration of 0.5 mM and allowed to proceed for 5 min. The reactions were terminated with 10% formic acid (2:1, v/v), and the samples were dried at 95°C for 30 min to remove unfixed ^14^C. Pellets were resuspended in Milli‐Q water and 2 mL of scintillation cocktail (Ultima Gold XR, PerkinElmer, US), and radioactivity was measured using a scintillation counter (Tri‐Carb, PerkinElmer, US). The background counts (no RuBP) were subtracted from the raw readings to calculate the fixed ^14^C, which was then converted to carbon fixation rates. Data are presented as mean ± standard deviation (SD).

### 2.8 Propanediol Dehydratase (PduCDE) Enzymatic Assays

The PduCDE activity was measured by monitoring NADH oxidation at 340 nm, coupled with propionaldehyde formation via alcohol dehydrogenase (Lehman et al. 2017). Each 200 μL reaction contained 0.5 μg purified sample, 200 mM 1,2‐propanediol, 25 μg·mL^−1^ *Saccharomyces cerevisiae* alcohol dehydrogenase, 0.4 mM NADH, 50 mM KCl, 50 mM HEPES (pH 7.5), and 20 μM adenosylcobalamin (AdoB_12_). The reactions were carried out in 96‐well plates and initiated by the addition of AdoB_12_. Absorbance at 340 nm was monitored at 37°C using a microplate reader (BMG LABTECH). Standard curves were generated using NADH concentrations ranging from 0.1 to 1 mM to quantify NADH consumption.

### 2.9 SDS‐PAGE and Immunoblot Analysis

SDS‐PAGE and immunoblotting were performed as described previously (Li et al. 2020, 2024; Sun et al. 2022). Briefly, 20–40 μg total protein was loaded into each well. The protein samples were mixed with SDS‐PAGE loading buffer, heated at 99°C for 10 min, and separated on 17% SDS‐PAGE gels. The gels were stained with Coomassie Brilliant Blue for total protein visualisation. For immunoblotting, gel electrophoresis was performed at 95 V for 50 min, and the proteins were transferred onto polyvinylidene fluoride (PVDF) membranes (Cytiva Life Sciences). The membranes were probed with primary rabbit polyclonal antibodies against anti‐CsoS1 (Agrisera, catalogue no. AS14 2760; dilution 1:5000), anti‐CbbL (Agrisera; catalogue no. AS03 037; dilution 1:10,000), followed by incubation with horseradish peroxidase‐conjugated secondary anti‐rabbit IgG antibody (Agrisera, catalogue no. AS09602; dilution 1:10,000). Signal detection was performed using a chemiluminescence kit (Bio‐Rad), and images were acquired using an ImageQuant LAS 4000 system. Protein band intensities were quantified using Fiji (ImageJ) software (Schindelin et al. 2012).

### 2.10 Negative‐Staining Transmission Electron Microscopy

For electron microscopy, 5 μL of isolated BMCs were placed on carbon‐coated grids (Carbon Films on 300 Mesh Grids Copper, Agar Scientific, UK) and incubated for 40 s. The grids were then stained and washed with 60 μL 2% uranyl acetate. Excess stain was removed using filter paper, and the grids were air‐dried for at least 1 min before imaging. Imaging was performed using a FEI Tecnai G2 Spirit Bio TWIN transmission electron microscope equipped with a Gatan Rio 16 camera. To analyse the BMC shell diameters, 50 individual shell particles were randomly selected from electron micrographs. For each particle, the diameters were measured by drawing three diagonals from different angles through the center using ImageJ (Fiji) software (Schindelin et al. 2012), and the average of the three measurements was used as the final diameter.

### 2.11 Super‐Resolution Fluorescence Microscopy

The *S. typhimurium* LT2 cells were imaged using a ZEISS Elyra 7 microscope in the Lattice Structured Illumination Microscopy (SIM) imaging mode. Images were captured using a Plan‐Apochromat 63×/1.4 oil DIC M27 objective and an SBS LP 560 dual camera beam splitter, specifically set up to minimise the fluorescence crosstalk. This setup included two tracks, one for GFP excited at 488 nm laser (Zeiss Laser HR Diode 488 nm, 500 mW; operated at 5%–20% of 500 mW output), and the other for mCherry excited at 561 nm laser (Zeiss Laser HR DPSS 561 nm, 500 mW; operated at 10%–30% of 500 mW output), each detected by separate cameras. The cameras were calibrated prior to each imaging session to ensure alignment. For the 488 nm track, emission was separated using SBS LP 560 with emission filters BP420–480/BP495–550 and BP570–620 plus LP655; for the 561 nm track, emission was separated using SBS BP490–560/LP640 with emission filters BP420–480/BP570–630/LP740 and BP495–550/LP655. SIM illumination was performed with grating periods of 27.5 μm (488 nm) and 32 μm (561 nm), using a 13‐phase pattern. Images were acquired on a PCO.edge 4.2 CLHS sCMOS camera at 1280 × 1280 pixels and 16‐bit depth. Raw SIM datasets were reconstructed in Zeiss ZEN 3.0 (black edition) using the SIM^2^ module. Reconstruction/post‐processing parameters were kept consistent within each experiment and included SIM^2^ “weak” filtering for the 488 and 561 channels (live/fixed samples, respectively), 2× processing/output sampling, and the fast‐fit median filter. Channel alignment was performed in ZEN using the instrument calibration routine prior to each imaging session and applied after SIM^2^ reconstruction to generate registered two‐channel images for downstream analysis. For time‐series datasets, images were kept in raw intensity scaling. For sample preparation, 5 μL of bacterial culture from various growth conditions was placed on the growth agar pads. The cells were allowed to adhere to the surface of the agar blocks, which were then covered with coverslips for microscopy. For mCherry and GFP localization analysis, cells were pelleted and washed twice with PBS buffer, fixed with 4% paraformaldehyde solution (prepared with PBS buffer), and incubated at room temperature for 15 min before imaging. All images were captured from at least three different cultures. For time‐lapse imaging, cells on the growth agar pads were incubated under a microscope at a controlled temperature of 37°C. Imaging was performed every minute to track the dynamics of α‐CBs, Pdu BMCs, and hybrid BMCs.

Post‐capture, image processing, and editing were conducted using the ImageJ (Fiji) software (Schindelin et al. 2012). Colocalization analysis was performed using the Coloc2 plugin to generate Pearson's correlation coefficient R (Bolte and Cordelières 2006). A Pearson's *R*‐value close to 1 reflects reliable colocalization, whereas values near zero indicate uncorrelated fluorescence distributions. The number of puncta within each bacterium across the entire field of view was determined using a specific macro available from the public GitHub repository (https://github.com/Marien‐kaefer/Count‐objects‐within‐objects). To analyse the diffusive behaviour and trajectories of the α‐CBs, Pdu BMCs, and hybrid BMCs, raw images were registered for horizontal drift using the descriptor‐based series registration (2d/3d+T) plugin in ImageJ. Following this adjustment, particle tracking was conducted using the TrackMate plugin in ImageJ (Fiji) (Sun et al. 2019). Retrieved track data were analysed using bespoke MATLAB (Mathworks) scripts for diffusion coefficients and MSDs previously developed within the laboratory (Sun et al. 2019), modified from methods described in earlier studies (Ewers et al. 2005; Sbalzarini and Koumoutsakos 2005). MSD was calculated using the square displacement of the particle position over time. The diffusion coefficients were calculated by performing linear regression on the first six points of the MSD versus Tau plots under the assumption that particle motion is primarily Brownian during these shorter intervals. The behaviour observed at higher tau values suggests non‐Brownian diffusion, and the coefficient obtained is referred to as the “apparent diffusion coefficient.”

### 2.12 Bioinformatic Analysis

Protein sequences of interest were retrieved from the public databases UniProt and the Protein Data Bank (PDB). Sequence identity and similarity were determined using the BLASTP algorithm in MEGA version 6.0. The results were visualised and highlighted in red using ESPript 3.0 (https://espript.ibcp.fr/ESPript/ESPript/).

## 3 Results

### 3.1 McdAB Mediates the Distribution of Recombinant α‐CBs in *Salmonella* Without Affecting the Positioning of Native Pdu BMCs

**FIGURE 1:**
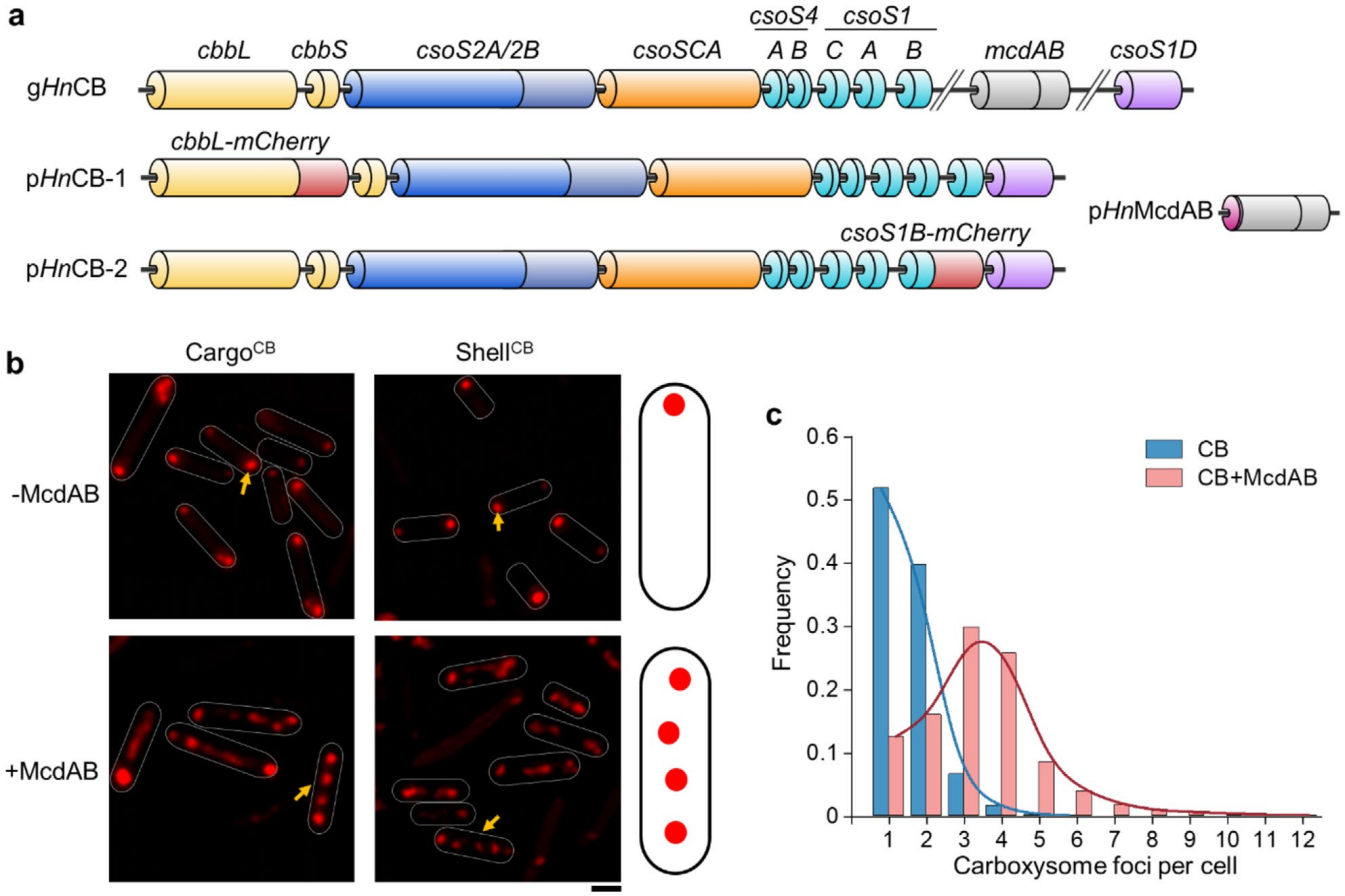
Expression of α‐CBs in *S. typhimurium* LT2 and proper distribution of α‐CBs mediated by McdAB. (a) Genetic organisations of the α‐CB *cso* operon from *H. neapolitanus* (g*Hn*CB), and the synthetic plasmids p*Hn*CB‐1 and p*Hn*CB‐2 for α‐CB visualisation, which fused the fluorescent protein mCherry to the C‐terminus of CbbL (Cargo^CB^) or CsoS1B (Shell^CB^), derived from the pBAD33‐CBS1D plasmid (Sun et al. 2022). The plasmid p*Hn*McdAB was used for McdAB expression, with a His‐tag at the N‐terminus of McdA. (b) Fluorescence images of wild‐type (WT) *S. typhimurium* LT2 expressing α‐CBs (Cargo^CB^ or Shell^CB^) exhibited polar aggregation in the absence of McdAB (‐McdAB) compared to multiple discrete mCherry foci in the presence of McdAB (+McdAB). Cells were grown in a microcompartment induction medium (MIM) with 0.5% succinate as the carbon source and induced with 1 mM arabinose. Scale bars, 1 μm. Yellow arrows indicate α‐CB particles discerned in the cell. Schematic models of the in vivo localization of α‐CBs are shown on the right. (c) Quantification of α‐CB foci per cell. With the mediation of McdAB, the number of α‐CBs in cells increased compared to polar aggregation in cells expressing α‐CBs alone. Mean of α‐CBs numbers per cell in the strain expressing CB: 1.59 ± 0.71 (mean ± SD, *n* = 1638); CB + McdAB: 3.32 ± 1.59 (mean ± SD, *n* = 1949). n represents the total number of cells analysed, combining cargo labelled and shell labelled cells (Cargo^CB^ and Shell^CB^).

The genes encoding α‐CB proteins are mainly clustered within the single *cso* operon in the genome of the chemoautotrophic bacterium *Halothiobacillus neapolitanus* ( *H. neapolitanus* ) (Figure 1a). In the previous work, we successfully generated recombinant α‐CBs in *Escherichia coli* ( *E. coli* ) to express the shell proteins (CsoS1A/B/C/D and CsoS4A/B), the scaffolding protein CsoS2, the large and small subunit of Rubisco (CbbL and CbbS), and β‐carbonic anhydrase (CsoSCA) (Sun et al. 2022). To construct α‐CBs in *S. typhimurium* LT2 and visualise their subcellular distribution, we created the plasmids p*Hn*CB‐1 and p*Hn*CB‐2, in which the fluorescent protein mCherry was fused to the C‐terminus of either CbbL (termed Cargo^CB^) or CsoS1B (termed Shell^CB^) (Figure 1a). Super‐resolution fluorescence imaging using a ZEISS Elyra 7 microscope in the Lattice Structured Illumination Microscopy (SIM) imaging mode revealed that the expression of p*Hn*CB‐1 and p*Hn*CB‐2 induced by arabinose resulted in the polar aggregation of α‐CBs (Figure 1b), and the distribution pattern of α‐CBs was consistent with those observed in *E. coli* and *Corynebacterium glutamicum* (Bonacci et al. 2012; Baumgart et al. 2017; Flamholz et al. 2020). In contrast, in the absence of α‐CBs, mCherry‐tagged CbbL and CsoS1B were evenly distributed throughout the cytosol (Figure S1). These results suggest that the formed α‐CBs were clustered only in the polar regions of *S. typhimurium* LT2 cells, likely owing to the absence of the cognate McdAB (Maintenance of Carboxysome Distribution protein A and B) system, which is essential for the proper positioning of α‐CBs in cells (MacCready et al. 2021).

To establish the subcellular positioning of heterologously expressed α‐CBs, we introduced McdAB proteins from *H. neapolitanus* into *S. typhimurium* LT2 cells (Figure 1a). When *S. typhimurium* LT2 cells co‐expressed α‐CBs and McdAB, both Cargo^CB^ and Shell^CB^ formed multiple discrete fluorescent foci within the cells (Figure 1b,c), resembling the typical BMC distribution observed in their native hosts (MacCready and Vecchiarelli 2021). This distribution was further confirmed by thin‐section electron microscopy (EM) of *S. typhimurium* LT2 cells expressing α‐CBs without fluorescence tagging (Figure S2a,b). Time‐lapse fluorescence imaging revealed that McdA oscillated along the longitudinal axis of cells, whereas α‐CBs appeared to colocalize with McdB and were mobile within their local regions (Figure S3, Videos S1 and S2), consistent with the α‐CB distribution in *H. neapolitanus* (MacCready et al. 2018, 2021).

It was suggested that the role of McdAB extends beyond carboxysome positioning, and ATP‐driven McdAB‐like systems may also determine the spatial organisation of metabolosomes (MacCready et al. 2021; MacCready and Vecchiarelli 2021). We assessed whether the engineered McdAB system would affect the positioning of native Pdu BMCs in *S. typhimurium* LT2. Genes expressing Pdu BMC proteins are clustered in a chromosomal *pdu* operon (Figure S4a). The addition of 1,2‐propanediol (1,2‐PD) can induce the *pdu* operon expression, leading to the formation of Pdu BMCs (Parsons et al. 2010; Cheng et al. 2011; Yang et al. 2022). To visualise Pdu BMCs, we used previously constructed GFP fusion strains (LT2‐*pduE‐sfGFP*, LT2‐*pduA‐sfGFP*, LT2‐*pduT‐eGFP*), in which the cargo protein PduE and shell proteins PduA or PduT were fused with GFP (Yang et al. 2022). These strains were grown in a microcompartment induction medium (MIM) supplemented with 1,2‐PD, in the presence or absence of McdAB. Fluorescence microscopy revealed that Pdu BMCs exhibited typical discrete puncta within the cells, regardless of the presence of McdAB (Figure S4b), indicating that the engineered McdAB did not affect the in vivo positioning of Pdu BMCs.

We further examined whether other ATP‐driven positioning systems in *S. typhimurium* LT2 affected Pdu BMC positioning. Specifically, ParA and MinD mediate plasmid/chromosome partitioning and division‐site placement, respectively (Pulianmackal et al. 2023). Our results revealed that Pdu BMCs remained discrete patches in the Δ*parA* and Δ*minD* mutants, similar to the distribution observed in the wild‐type (WT) strain (Figure S4c). These findings indicate that neither endogenous ParA nor MinD plays a role in determining Pdu BMC positioning in *S. typhimurium* LT2.

Collectively, our results demonstrated that functional α‐CBs were robustly expressed in *S. typhimurium* LT2 using plasmids that integrate the *cso* operon and that the engineered McdAB system effectively mediated the positioning of recombinant α‐CBs in *S. typhimurium* LT2 cells without affecting the subcellular distribution of native Pdu BMCs.

### 3.2 Formation and Mobility of Hybrid BMCs Mediated by Exchange of Shell Proteins of α‐CBs and Pdu BMCs

**FIGURE 2:**
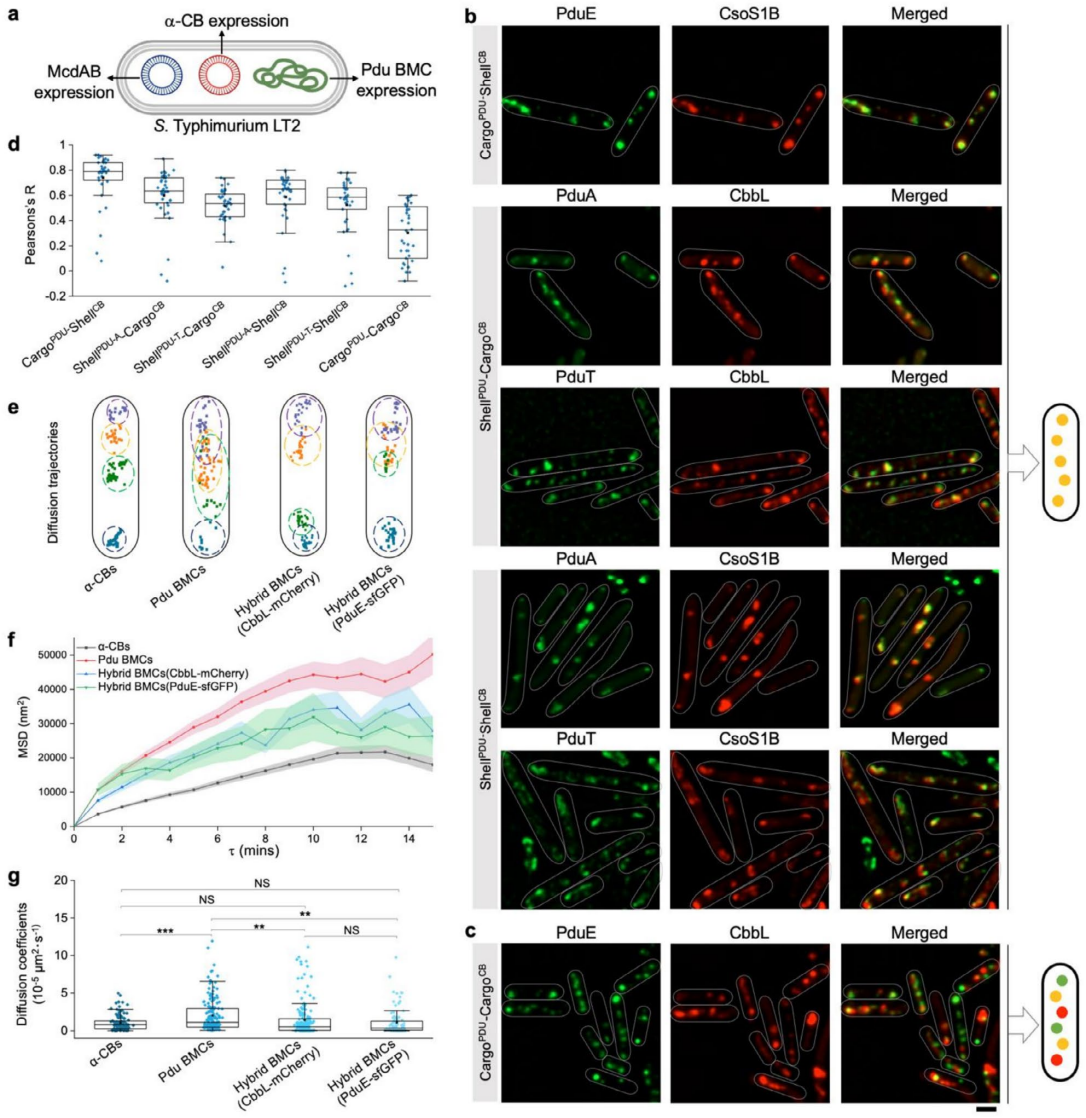
Hybrid BMCs were formed in cells expressing α‐CBs, Pdu BMCs, and McdAB. (a) Schematic of co‐expressing Pdu BMCs, α‐CBs, and McdAB in *S. typhimurium* LT2. (b,c) Fluorescence images showing localization of α‐CB and Pdu BMC shell/cargo proteins in cells grown in MIM with 1,2‐PD and induced with 1 mM arabinose. Cargo^PDU^ represents PduE‐sfGFP, Cargo^CB^ represents CbbL‐mCherry, Shell^PDU^ represents PduA‐sfGFP and PduT‐eGFP, and Shell^CB^ represents CsoS1B‐mCherry. Scale bar: 1 μm. Schematic models on the right summarise shell and cargo distribution. (d) Colocalization analysis of GFP and mCherry in (b) and (c) (*n* = 38). The Pearson's R values for all strains are: 0.74 ± 0.19 (Cargo^PDU^‐Shell^CB^); 0.60 ± 0.21 (Shell^PDU‐A^‐Cargo^CB^); 0.52 ± 0.14 (Shell^PDU‐T^‐Cargo^CB^); 0.59 ± 0.21 (Shell^PDU‐A^‐Shell^CB^); 0.52 ± 0.23 (Shell^PDU‐T^‐Shell^CB^); 0.30 ± 0.22 (Cargo^CB^‐Cargo^PDU^). Data are represented as mean ± SD. Boxplots show median (line), mean (square), and whiskers within 1.5 times interquartile range. (e) Diffusion tracking of α‐CBs, Pdu BMCs, and hybrid BMCs over 60 min (1‐min interval). Pots and circles indicate the mobility trajectories and regions of individual BMCs. (f) Mean square displacement (MSD) vs. time interval (τ) profiles showing mobility of each BMC type. Each coloured line and symbol represent the MSD of τ over time for different groups. (g) Analysis of apparent diffusion coefficient across different BMC types over 60 min. Average diffusion coefficient per BMC: 1.07 × 10^−5^ μm^2^·s^−1^ (*n* = 118) for α‐CBs, 2.05 × 10^−5^ μm^2^·s^−1^ (*n* = 167) for Pdu BMCs, 1.43 × 10^−5^ μm^2^·s^−1^ (*n* = 170) for hybrid BMCs with CbbL‐mCherry and 1.10 × 10^−5^ μm^2^·s^−1^ (*n* = 92) for hybrid BMCs with PduE‐sfGFP. Two‐tailed unpaired *t* test, ***p < 0.001; **p < 0.01; NS, *p* > 0.05. n represents the number of cells.

The generation of *S. typhimurium* LT2 mutants that can synthesise both Pdu BMCs and α‐CBs, along with the introduction of proper positioning systems, provides a framework for deciphering the physical and physiological coordination of two different types of BMCs within a single cell. To study the in vivo assembly and subcellular localization of both Pdu BMCs and α‐CBs, we expressed α‐CBs and McdAB in *S. typhimurium* LT2 strains (Figure 2a), followed by super‐resolution fluorescence imaging and colocalization analysis. The results revealed the colocalization of PduE‐sfGFP and CsoS1B‐mCherry (Cargo^PDU^‐Shell^CB^), PduA‐sfGFP/PduT‐eGFP and CbbL‐mCherry (Shell^PDU^‐Cargo^CB^), as well as PduA‐sfGFP/PduT‐eGFP and CsoS1B‐mCherry (Shell^PDU^‐Shell^CB^) (Figure 2b,d), indicating the interchangeability of Pdu BMC and α‐CB proteins and the formation of hybrid BMCs. Interestingly, hybrid BMCs were also formed in the absence of McdAB: PduE‐sfGFP and CsoS1B‐mCherry (Cargo^PDU^‐Shell^CB^) formed multiple clusters from the middle to one side of the cell (Figure S5a), strikingly distinct from the typical distribution of Pdu BMCs (Figure S4c) and single polar puncta of α‐CB without McdAB (Figure 1b). These observations were further supported by thin‐section EM results of multiple cells from independent preparations (Figure S2).

In contrast, PduE‐sfGFP showed a relatively low level of colocalization with CbbL‐mCherry (Cargo^PDU^‐Cargo^CB^), regardless of the presence or absence of McdAB (Figures 2c,d and S5b,d), suggesting limited interchangeability between Pdu and α‐CB cargo enzymes in the WT background. To validate this observation, we conducted fluorescence colocalization analysis in the Δ*pduK* *S. typhimurium* LT2 strain. As *pduK* deficiency could induce polarised clustering of Pdu BMCs (Yang et al. 2022), the reduction in the number of fluorescence foci resulted in a less complex spatial landscape, thereby facilitating accurate colocalization analysis. Fluorescence imaging revealed that PduE‐sfGFP and CbbL‐mCherry (Cargo^PDU^‐Cargo^CB^) rarely colocalized (Figure S5c,d), suggesting that Pdu and α‐CB cargo enzymes remained physically segregated by the formed BMC structures, likely owing to the intrinsic specificity of the biogenesis and encapsulation mechanisms of distinct BMCs (Melnicki et al. 2021).

We then examined whether the mixture of shell proteins from different origins will influence the in vivo mobility of the formed hybrid BMCs by time‐lapse imaging of PduE‐sfGFP and CbbL‐mCherry. Mean square displacement (MSD) analysis showed that native Pdu BMCs exhibited larger mobility regions in *S. typhimurium* LT2 cells than α‐CBs, whereas hybrid BMCs displayed intermediate mobility regions between Pdu BMCs and α‐CBs (Figure 2e,f; Video S2). Moreover, hybrid BMCs and α‐CBs had comparable mean diffusion coefficients, both lower than that of Pdu BMCs (Figure 2g), indicating that the exchange of shell proteins altered the mobility of hybrid BMCs compared to α‐CBs and Pdu BMCs on their own. Consistently, McdAB was shown to determine the positioning of Pdu‐based hybrid BMCs in the PduK‐free background, suggesting the presence of α‐CB shell proteins that interact with McdB in the formed hybrid BMCs (Figure S5c).

### 3.3 Interchangeability of Individual BMC Shell Proteins

**FIGURE 3:**
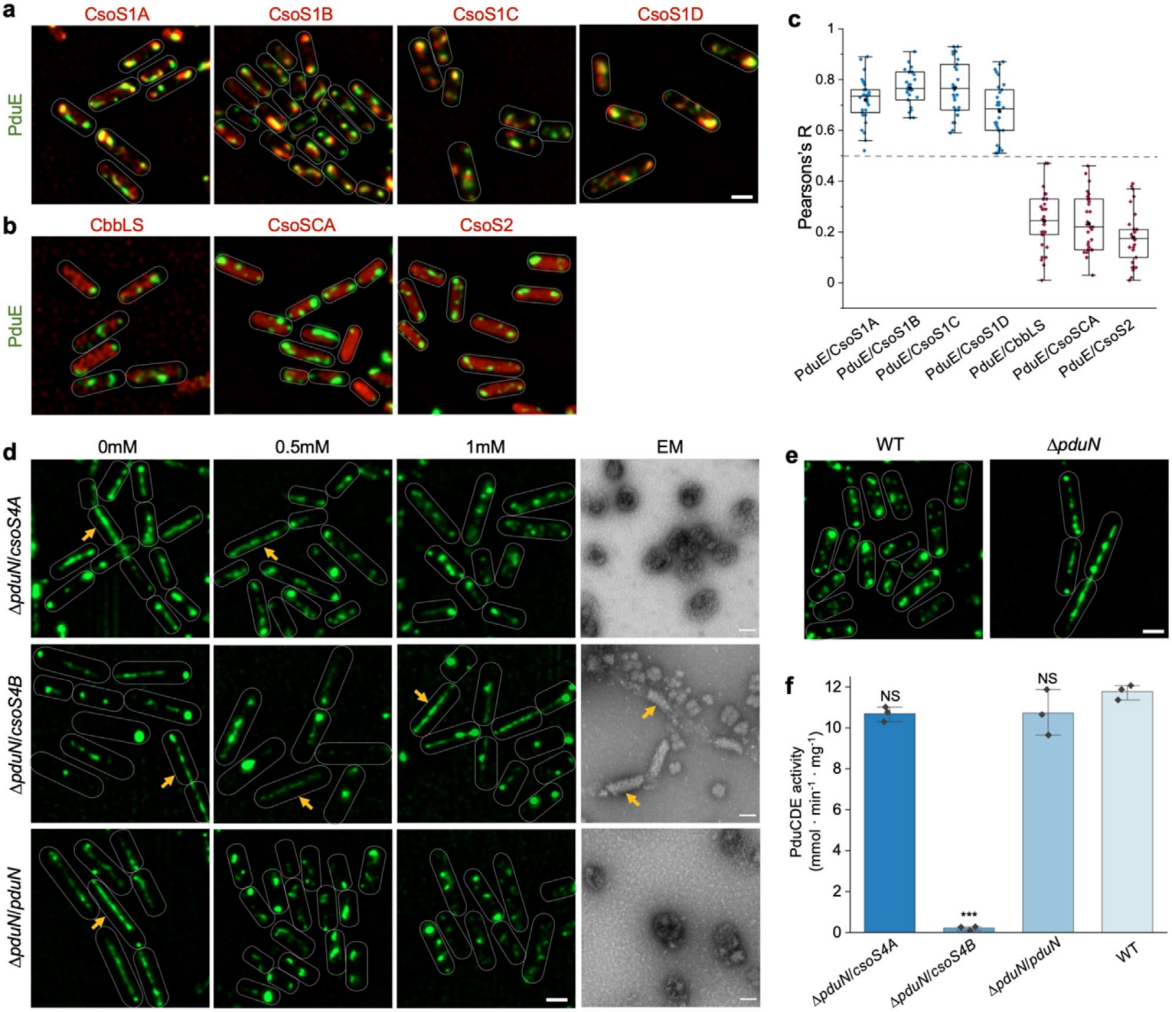
Interchangeability of individual components between α‐CBs and Pdu BMCs. (a,b) Fluorescence image of LT2‐*pduE‐sfGFP* strain harbouring pBAD33 plasmid expressing individual α‐CB proteins tagged with mCherry grown in MIM with 1,2‐PD and induced with arabinose. Scale bar: 1 μm. (c) Colocalization analysis of sfGFP and mCherry in (a) and (b). The Pearson's R values for these strains were: 0.72 ± 0.08 (PduE/CsoS1A); 0.76 ± 0.06 (PduE/CsoS1B); 0.77 ± 0.10 (PduE/CsoS1C); 0.678 ± 0.11 (PduE/CsoS1D); 0.24 ± 0.11 (PduE/CbbLS); 0.23 ± 0.11 (PduE/CsoSCA) and 0.18 ± 0.10 (PduE/CsoS2). Data are presented as mean ± SD for *n* = 30 cells. (d) Fluorescence images of Δ*pduN* strain supplemented with a modified IPTG‐inducible plasmid pBAD33, expressing CsoS4A, CsoS4B, or PduN with an additional visualisation vector PduE‐sfGFP. Strains were grown in MIM supplemented with 1,2‐PD and induced with different concentrations of IPTG. TEM images show purified BMC samples from Δ*pduN* strains grown with 1 mM IPTG induction without fluorescent vector. Yellow arrows indicate elongated structures. Scale bars: 1 μm for fluorescence images and 100 nm for TEM images. (e) Fluorescence image of WT *S. typhimurium* LT2 and Δ*pduN* strain carrying PduE‐sfGFP vector grown in MIM with 1,2‐PD. Scale bar, 1 μm. (f) PduCDE (diol dehydratase) activity of purified Pdu BMCs from WT and Δ*pduN* complemented with CsoS4A, CsoS4B or PduN. The PduCDE activity was normalised by the total protein amount of the samples. Statistical comparisons of all samples with WT were performed to quantify differences in enzyme activity. Two‐tailed unpaired *t* test, ***p < 0.001. NS, *p* > 0.05. The center for error bars represents the mean, and error bars represent SD. *n* = 3 biologically independent experiments.

To systematically evaluate the interchangeability of individual components from Pdu BMCs and α‐CBs, we generated a collection of plasmids to express individual α‐CB proteins fused with mCherry in the LT2‐*pduE‐sfGFP* strain. Without Pdu BMC formation (no 1,2‐PD induction), all α‐CB proteins exhibited cytosolic distribution, with occasional aggregations observed at the cell poles (Figure S1), although CsoS1C formed more polar aggregates than other α‐CB proteins. When Pdu BMCs were present (induced by 1,2‐PD), the α‐CB shell proteins (CsoS1A, CsoS1B, CsoS1C, CsoS1D) colocalized with PduE‐sfGFP and displayed typical Pdu BMC distribution (Figure 3a,c). These results suggest that α‐CB shell hexamers were efficiently integrated into Pdu BMC shells, likely due to the high sequence similarity among BMC shell hexamers (Figure S6a,b).

In contrast, the α‐CB linker protein (CsoS2) and cargo proteins (CbbLS and CsoSCA) did not colocalize with PduE‐sfGFP and remained diffusive throughout the cytosol (Figure 3b,c), indicating that these proteins were unable to integrate into Pdu BMCs when their cognate shell proteins were not present in the shell at proper proportions and organizations. These findings highlight the distinct encapsulation mechanisms exploited by different BMCs (Li et al. 2020, 2024; Yang et al. 2022).

Notably, the pentameric shell proteins, CsoS4A and CsoS4B, displayed an even cytosolic distribution when Pdu BMCs were present (Figure S7a). This is likely attributed to their relatively low sequence similarity to the Pdu shell pentamer PduN (32.93% and 28.4%, respectively; Figure S6c) and the fact that only up to 12 pentamers are required to form an intact BMC shell (Tanaka et al. 2008; Sun et al. 2019, 2022; Yang et al. 2020; Sun, Chen, et al. 2025; Sun, Sheng, et al. 2025). To further evaluate the functional incorporation of α‐CB shell pentamers into Pdu BMCs, we overexpressed CsoS4A, CsoS4B, or PduN, together with PduE‐sfGFP, in the Δ*pduN S. typhimurium* LT2 strain (Yang et al. 2022; Figure 3d). Without PduN, Pdu BMCs displayed elongated structures, in contrast to the Pdu BMC puncta observed in WT cells (Figure 3e). Super‐resolution fluorescence imaging showed that IPTG‐induced expression of either CsoS4A or PduN in the Δ*pduN* strain suppressed the formation of elongated structures and enhanced punctate distribution of BMCs (Figure 3d). Interestingly, the overexpression of CsoS4B had limited effects on the elongated morphology of Pdu BMCs in the Δ*pduN* strain (Figure 3d).

To validate the in vivo fluorescence imaging results, we purified the synthesised BMCs from the Δ*pduN* strains that express CsoS4A, CsoS4B, or PduN (Figure S7b). EM revealed that the morphologies of the isolated BMCs were consistent with those determined by fluorescence imaging (Figure 3d). Enzymatic assays further demonstrated that either CsoS4A or PduN alone restored the functionality of Pdu BMCs lacking PduN to levels comparable to native Pdu BMCs, while CsoS4B did not recover the PduCDE (diol dehydratase) activity of the formed Pdu BMCs (Figure 3f). Together, these results imply that compared to the CsoS4A and PduN pentameric proteins that cap the vertices of polyhedral BMC shells, CsoS4B may have different roles in shaping BMC structures and functions.

### 3.4 in vitro Characterisation of Formed Hybrid BMCs

Fluorescence imaging demonstrated the formation of hybrid BMCs by co‐expressing Pdu BMCs and α‐CBs with McdAB in *S. typhimurium* LT2. To assess the structure and function of hybrid BMCs, we purified the formed hybrid BMCs (without fluorescent tagging) from *S. typhimurium* LT2. Following the established purification procedures specific for α‐CBs and Pdu BMCs (Li et al. 2020; Yang et al. 2020), we isolated two groups of hybrid BMCs: hybrid BMCs‐1 (following the α‐CB purification procedure) and hybrid BMCs‐2 (following the Pdu BMC purification procedure).

**FIGURE 4:**
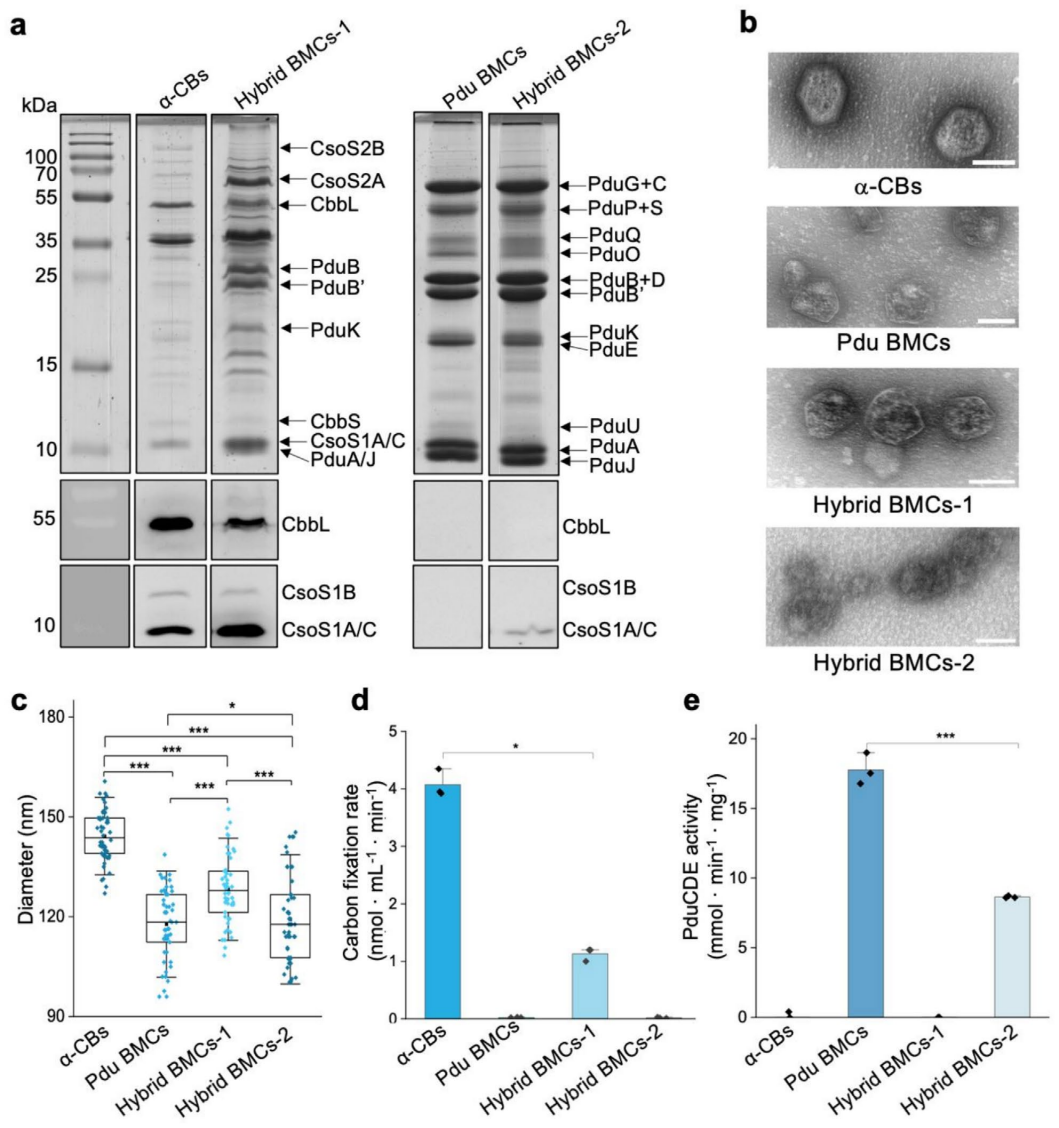
in vitro purification, protein detection, and enzyme activity analysis for hybrid BMCs. (a) SDS‐PAGE and immunoblots of purified samples from α‐CBs, Pdu BMCs, and hybrid BMCs. α‐CBs and hybrid BMCs‐1 were purified based on α‐CBs purification protocol, and purified samples were collected from 40% sucrose fractions. Pdu BMCs and hybrid BMCs‐2 were purified based on Pdu BMCs purification protocol. The control Pdu BMCs‐1 purified following the α‐CB purification procedure is shown in Figure S8. (b) TEM images of purified α‐CBs, Pdu BMCs, and hybrid BMCs. Images are representative of multiple samples obtained from independent preparations. All scale bars represent 100 nm. (c) Diameter of BMC size from purified α‐CBs, Pdu BMCs, and hybrid BMCs. The sizes of individual BMCs were: 144.22 ± 7.75 nm (α‐CBs), 117.75 ± 10.64 nm (Pdu BMCs), 128.23 ± 10.23 nm (Hybrid BMCs‐1), and 119.22 ± 12.94 nm (Hybrid BMCs‐2). Data are presented as means ± SD. *n* = 50. (d) Carbon fixation rates of purified α‐CBs, Pdu BMCs and hybrid BMCs (*n* = 3) were normalised by the total protein amount of the samples. The hybrid BMCs‐1 had decreased Rubisco activity than α‐CBs (*p* < 0.05, *n* = 3). The fixation rates per Rubisco were normalised by the Rubisco contents (Figure S9). (e) PduCDE activity of purified α‐CBs, Pdu BMCs, and hybrid BMCs. The hybrid BMCs‐2 had a 2‐fold decrease in PduCDE activity compared to Pdu BMCs (*p* < 0.001, *n* = 3). The PduCDE activity was normalised by the total protein amount of the samples. Two‐tailed unpaired *t* test, *p < 0.05; ***p < 0.001.

SDS‐PAGE analysis revealed that hybrid BMCs‐1 mainly contained α‐CB proteins (CsoS1, CsoS2, CbbL, CbbS) as well as several proteins possibly corresponding to Pdu shell components (PduA/B/B′/J/K; Figure 4a, left). Conversely, hybrid BMCs‐2 primarily comprised Pdu proteins, and their SDS‐PAGE profiles resembled those of native Pdu BMCs (Figure 4a, right). As a control, we purified BMCs from cells that express only Pdu BMCs following the α‐CB purification procedure (Pdu BMCs‐1; Figure S8). The major Pdu protein bands were substantially weaker in this control compared to the sample from the Pdu BMC purification procedure. This indicates that the α‐CB purification procedure could not enrich Pdu BMCs that lack α‐CB shell proteins. Immunoblot analysis confirmed the presence of CsoS1A/C in both hybrid BMCs‐1 and hybrid BMCs‐2, but not in the Pdu‐only controls purified following either procedure (Figures 4a, and S8). These results rule out non‐specific co‐isolation caused by the purification procedure and, together with fluorescence imaging, demonstrate that the formed hybrid BMCs contain a mixture of shell components from both α‐CBs and Pdu BMCs. The minor components CsoS1B were not detected in the purified hybrid BMCs‐2, due to its relatively low abundance (Sun et al. 2022). Intriguingly, CbbL was detected in both α‐CBs and hybrid BMCs‐1, but not in Pdu BMCs and hybrid BMCs‐2. This indicates that hybrid BMCs‐2 obtained following the Pdu BMC purification procedure encapsulated only Pdu cargo proteins and appeared to be exclusive to α‐CB cargo proteins. Thus, we postulate that hybrid BMCs‐1 obtained following the α‐CB purification procedure encapsulated only α‐CB cargo proteins.

EM images showed that both hybrid BMCs‐1 and hybrid BMCs‐2 retained intact, polyhedral architectures (Figure 4b). Hybrid BMCs‐1 and hybrid BMCs‐2 had an average diameter of 128.23 ± 10.23 nm and 119.22 ± 12.94 nm, respectively, slightly larger than Pdu BMCs (117.75 ± 10.64 nm) but smaller than recombinant α‐CBs (144.22 ± 7.75 nm, Figure 4c). Rubisco activity assays revealed that hybrid BMCs‐1 exhibited decreased CO_2_‐fixing activities, whereas no Rubisco activity was detected for hybrid BMCs‐2 (Figures 4d and S9), which is in agreement with the absence of CbbL in hybrid BMCs‐2 (Figure 4a, right). Moreover, hybrid BMCs‐1 did not display PduCDE activity, suggesting that the Pdu cargo proteins were likely not sequestered within hybrid BMCs‐1. In contrast, hybrid BMCs‐2 exhibited PduCDE activity, though approximately twofold decrease compared to native Pdu BMCs (Figure 4e), which may be attributed to the alternations of Pdu BMC protein composition and stoichiometry resulting from the formation of hybrid BMCs. Attempts to isolate hybrids BMCs containing both Pdu and α‐CB cargo enzymes were unsuccessful, likely because cargo proteins remained physically sequestered in different hybrid BMCs and the purification methods optimised for specific BMCs appeared to selectively enrich BMC structures containing only their corresponding cargo enzymes. The reason why distinct purification methods yielded mainly specific types of hybrid BMCs merits further investigation.

## 4 Discussion

Self‐assembly and modularity of BMCs play crucial roles in facilitating autotrophic CO_2_ fixation and various catabolic processes. Understanding how thousands of distinct BMC components assemble in space and time to generate intact and functional entities is vital for modulating BMC construction and optimising metabolic activities in synthetic biology. Although the assembly and functions of α‐CBs and Pdu BMCs have been investigated individually (Havemann and Bobik 2003; Bonacci et al. 2012; Yang et al. 2020; Sun et al. 2022; Ni et al. 2023; Wang et al. 2024), their assembly processes when both are present within a single host cell have not been well characterised. In this work, we developed a system using *S. typhimurium* LT2 to co‐express native Pdu BMCs and non‐native α‐CBs along with their positioning system, and extensively dissected the structural and functional interplays of α‐CBs and Pdu BMCs within the same cell using super‐resolution fluorescence microscopy, electron microscopy, and biochemical and enzymatic assays.

**FIGURE 5:**
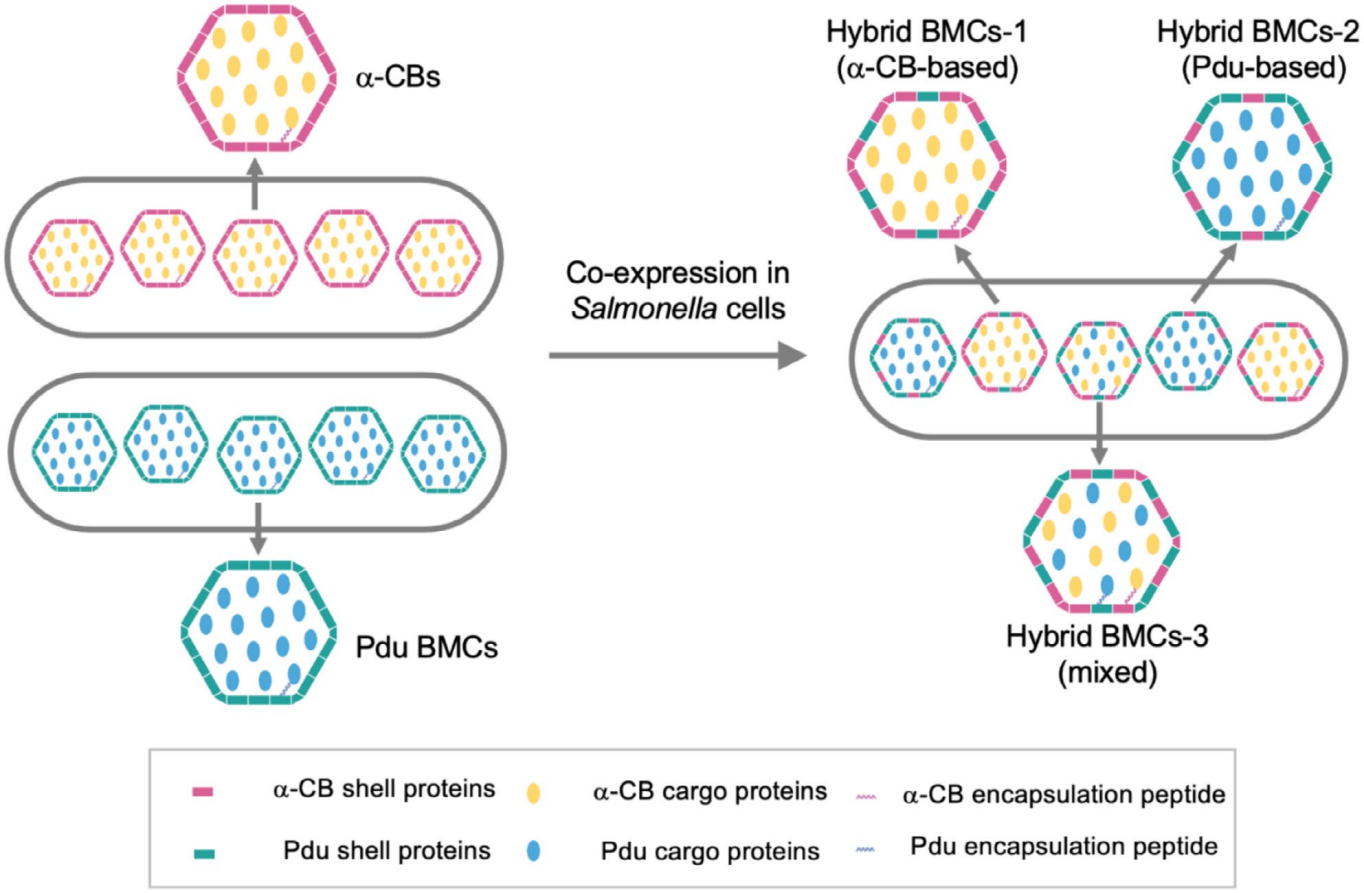
Model of BMC organisation in cells expressing either a single type or two types of BMCs. Cells expressing only one type of BMC, either α‐CBs or Pdu BMCs, encapsulate their respective cargo enzymes within their shell proteins. Co‐expression of α‐CBs and Pdu BMCs leads to the formation of three types of hybrid BMCs with mixed shells composed of both α‐CB and Pdu shell proteins. Hybrid BMC‐1 contains α‐CB cargos and a shell mainly composed of α‐CB shell proteins with some Pdu shell components. Hybrid BMC‐2 contains Pdu cargos and a shell mainly composed of Pdu shell proteins with some α‐CB shell components. Hybrid BMC‐3 is a putative type that might contain both α‐CB and Pdu cargos within a single shell. Cargo encapsulation is mediated by encapsulation peptides specific to the α‐CB and Pdu systems. The protein stoichiometry illustrated in the model does not reflect the actual stoichiometry of formed BMCs.

Our in vivo findings show that co‐expression of α‐CB and Pdu BMC proteins in *S. typhimurium* LT2 cells led to hybrid BMC formation, predominantly through exchange of shell proteins (Figure 5). Specifically, we found that most shell proteins from α‐CBs (except for CsoS4B) were integrated into Pdu BMCs during BMC biogenesis, whereas the cargo and linker proteins of α‐CB and Pdu BMCs remained predominantly segregated within individual hybrid BMCs. Alternations in shell protein composition further resulted in different mobility behaviours of hybrid BMCs compared to their parental BMCs in cells. in vitro analysis further revealed two types of hybrid BMCs, each comprising a mixture of α‐CB and Pdu shell proteins and encapsulating cargo enzymes specific for either CO_2_ fixation (α‐CB‐based hybrid BMCs‐1) or 1,2‐PD degradation (Pdu‐based hybrid BMCs‐2), but not both within the same BMC structure (Figure 5). Interestingly, our fluorescence imaging data occasionally showed rare colocalization events of cargo proteins from different BMC origins (Figure S5b,c). This potentially indicates a third, putative type of hybrid BMCs (hybrid BMCs‐3), in which both Pdu and α‐CB cargo enzymes are encapsulated by a shell with mixed Pdu and α‐CB shell proteins. Future studies developing biochemical purification and employing quantitative proteomics and structural analysis will be required to validate the presence and provide more detailed stoichiometric and functional characterizations of these hybrid BMCs (Yang et al. 2020; Sun et al. 2022; Sun, Chen, et al. 2025; Sun, Sheng, et al. 2025).

We found that both hybrid BMCs‐1 and hybrid BMCs‐2 exhibited reduced enzymatic activities compared to their parental compartments (Figure 4). This reduction likely arises from multiple factors: (i) the mixed shell composition may alter central pore properties, thereby influencing substrate permeability (Chowdhury et al. 2015; Park et al. 2017; Faulkner et al. 2020; Sarkar et al. 2024); (ii) the compositional changes may disturb the stoichiometric balance between shell proteins and cargo‐binding scaffolds, which is known to be critical for efficient cargo encapsulation and functionality (Oltrogge et al. 2020; Sun et al. 2022). Together, these factors likely account for the diminished catalytic activities of the hybrid BMCs and highlight the precise tuning of shell‐cargo interactions in native BMCs.

Our observations align with prior work showing that co‐expression of Pdu and Eut BMCs in *Salmonella* produced hybrid microcompartments with functional deficits, which likely explains the mutually exclusive transcriptional regulation of these operons (Sturms et al. 2015). Additionally, the early study revealed that ectopic expression of individual non‐cognate shell proteins disrupted Pdu BMC function, suggesting that promiscuous shell interactions impose remarkable functional costs during dual‐system expression (Sturms et al. 2015). The extensive shell exchange, largely segregated cargo organisation, and altered mobility observed collectively in our work revealed how conserved shell interfaces and system‐specific encapsulation rules act as organising principles that help maintain BMC identity and function in vivo.

BMCs are modular and the shell proteins are generally conserved across different BMCs, providing a framework for the construction of chimeric organelles. For example, previous work has explored replacement or integration of β‐CB shell proteins with α‐CB or Pdu homologues (Cai et al. 2015; Fang et al. 2018). However, any modification of shell components and key interacting residues may alter shell assembly and permeability (Cai et al. 2015) or disrupt crystal packing contacts and orientation (Pang et al. 2014; Kerfeld and Erbilgin 2015; Slininger Lee et al. 2017). Moreover, in native hosts, BMC structures are morphologically heterogeneous and vary in size and shape depending on environmental conditions (Sun et al. 2019, 2022; Yang et al. 2020). As such, targeted incorporation of shell proteins from different BMC types and organisms into one BMC structure with specific functions still remains a challenging benchmark. Our systematic study revealed that individual α‐CB shell proteins from *H. neapolitanus* could be integrated into native Pdu BMCs (Figure 3). This integration probably relies on the conserved structural motifs such as KAA and PRPH, which promote self‐assembly through shape complementarity, hydrogen bonds, and conserved amino acids at the edge interface (Sutter et al. 2017; Melnicki et al. 2021; Trettel et al. 2024). Interestingly, we found that the BMC‐T protein CsoS1D could be incorporated into Pdu BMCs (Figure 3a). In contrast, BMC‐T derived from *Haliangium ochraceum* exhibited no detectable capacity for integration into β‐CBs from the cyanobacterium *Synechococcus elongatus* PCC 7942 (MacCready et al. 2024). These findings suggest that BMC‐T proteins from different BMC systems have subtle structural specificity and conserved interaction interface with the “assembler”, which allow them to be incorporated into non‐native BMC shells (Roberts et al. 2012; Lassila et al. 2014; Greber et al. 2019). Our results extend this shell‐swapping paradigm to a cross‐class pairing of native Pdu BMCs and heterologous α‐CBs, and provide in vivo evidence for extensive shell intermixing between distinct BMC types through conserved interface motifs while cargo recruitment remains largely BMC‐specific.

From an applied perspective, our findings highlight shell composition may be a tunable design parameter for engineering BMC‐based metabolic modules. Since the pore structural properties and local physicochemical environment of major shell proteins govern the transport of substrate and product molecules across the shell (Kerfeld et al. 2018; Faulkner et al. 2020; Liu 2022), controlled alteration of non‐cognate shell components may enable rational manipulation of pore size, electrostatics, and inter‐protein interfaces to fine‐tune substrate access and product release to meet pathway‐specific requirements, thereby providing new avenues for tailoring BMC architecture and function in future microbial biotechnological applications.

Notably, only CsoS4A, and not its isoform CsoS4B, was able to substitute for PduN (Figure 3d,f), likely due to their structural differences. CsoS4A has a wider pore and overall architecture compatible with vertex capping. In contrast, CsoS4B possesses a narrower pore, distinct loop flexibility, and altered surface electrostatics (Tanaka et al. 2008; Zhao et al. 2019), which may reduce its ability to functionally replace PduN. These findings suggest the specialised roles of distinct pentamer isoforms, with implications for BMC evolution and the design of synthetic compartments.

Without α‐CB shell proteins in the shell, individual α‐CB scaffold and cargo proteins (CsoS2, Rubisco, CsoSCA) could not be integrated into Pdu BMCs (Figure 3b). This is likely because cargo recruitment relies on specialised encapsulation peptides (EPs) that link the shell and cargo proteins. For example, in α‐CBs, CsoS2 interacts with Rubisco via its N‐terminal region and binds to shell proteins through its middle and C‐terminal regions (Oltrogge et al. 2020; Ni et al. 2023; Wang et al. 2024). Similarly, the N‐terminal extension of PduD in Pdu BMCs serves as EPs, facilitating the recognition and loading of PduCDE (Fan and Bobik 2011; Yang et al. 2022). Therefore, integrating cognate encapsulation systems is an important consideration in synthetic engineering to achieve efficient cargo loading within recombinant or chimeric BMCs. In addition to the use of endogenous EPs, recent studies have indicated that the construction of engineered BMCs for defined functions requires the rational design of EPs compatible with both target cargo and shell proteins, thereby enhancing the recognition and encapsulation of non‐native cargos (Li et al. 2024; Sun, Chen, et al. 2025; Sun, Sheng, et al. 2025).

In this work, live‐cell lattice SIM^2^ imaging was employed for dual‐colour colocalization and mobility measurements. Although SIM cannot analyse single‐molecule stoichiometry, it is sufficient to assess in vivo hybrid BMC organisation and dynamic behaviours at a high resolution. Future work using localization‐based approaches, such as PALM or STORM, will enable direct determination of the molecular stoichiometry of hybrid BMCs. Additionally, chromosomal integration of the α‐CB genes in *S. typhimurium* LT2 would also provide tighter expression control to enhance the robustness and physiological integration of heterologous expression systems for rational design and bioengineering applications.

Proper intracellular positioning of BMCs is thought to be vital for efficient function and inheritance by daughter cells (Pulianmackal et al. 2023). Among the known positioning systems, ParA/MinD ATPases are responsible for spatially organising a variety of cellular structures such as diverse protein‐based organelles (MacCready and Vecchiarelli 2021), divisomes (Lutkenhaus 2012), and chromosomes (Havey et al. 2012). The McdAB system spatially organises α‐CBs in *H. neapolitanus* and β‐CBs in *Synechococcus elongatus* PCC 7942 along the nucleoid (MacCready et al. 2018, 2021). Our study demonstrates that functional α‐CBs can be constructed and properly positioned in the pathogenic bacterium *Salmonella* by introducing its cognate McdAB. This has implications for engineering functional BMCs in heterologous hosts while maintaining native regulatory control and heritable organelle positioning.

Although several BMC operons encode putative McdAB systems (Axen et al. 2014), not all of them exploit McdAB‐like positioning. For instance, our results show that neither McdAB nor ParA/MinD affected the localization of Pdu BMCs in *S. typhimurium* LT2 (Figure S4). One explanation might involve the evolutionary divergence of the functions of these proteins, since McdA‐like proteins responsible for BMC distribution are typically encoded within or near their BMC operon (MacCready et al. 2021; MacCready and Vecchiarelli 2021). In contrast, the *parA* gene is located on the pSLT plasmid of *S. typhimurium* LT2 and *minD* is located far from the *pdu* operon (McClelland et al. 2001). Further studies are required to elucidate the positioning mechanisms of Pdu BMCs in *Salmonella*.

Metagenomic analyses have shown that approximately 22% of the BMC‐containing genomes encode two or more loci (Sutter et al. 2021). Organisms harbouring three or more BMC types are predominantly *Proteobacteria* or *Firmicutes*, particularly within the *Enterobacterales* or *Clostridiales* orders (Sutter et al. 2021). Many of these organisms are pathogenic to humans and other animals and are abundant in aquatic and soil environments (Fendrich et al. 1990; Sutter et al. 2021). The ability to produce multiple functionally diverse BMCs confers metabolic versatility and enables these organisms to thrive in challenging environments. In this context, our study, although introducing non‐native CBs, establishes a foundation to probe the modular assembly of different BMC types within a single cell and the exact rules that mediate the construction of functionally diverse BMCs. These results have implications for how pathogenic microorganisms naturally encode multiple BMC types within their genomes and maintain diverse BMCs to work together. It also paves the way for modular design and bioengineering of BMC‐based nanostructures for new metabolic functions.

## Author Contributions

**Marie Held:** resources, methodology, writing – review and editing. **Tianpei Li:** methodology, formal analysis, writing – review and editing. **Lu‐Ning Liu:** conceptualization, supervision, formal analysis, funding acquisition, project administration, writing – review and editing. **Yu Chen:** methodology, formal analysis, writing – review and editing. **Mengru Yang:** methodology, formal analysis, writing – review and editing. **Ping Chang:** investigation, data curation, validation, formal analysis, writing – original draft.

## Funding

This work was supported by the National Key R&D Program of China (2021YFA0909600, 2023YFA0914600), the Biotechnology and Biological Sciences Research Council (BB/Y01135X/1, BB/V009729/1, BB/Y008308/1), the Royal Society (URF\R\180030), and a University of Liverpool‐China Scholarship Council PhD studentship (to P.C.).

## Conflicts of Interest

The authors declare no conflicts of interest.

## Supporting information


**Figure S1:** Fluorescence microscopy for individual α‐CB proteins in the absence of α‐CBs in *S. typhimurium* LT2 cells.
**Figure S2:** Thin‐section electron microscopy (EM) of α‐CBs, Pdu BMCs and hybrid BMCs in *Salmonella* cells.
**Figure S3:** Subcellular localization of α‐CBs and McdA/McdB in *S. typhimurium* LT2 cells.
**Figure S4:** Assessment of McdAB, ParA, and MinD proteins on the spatial arrangement of Pdu BMCs in *Salmonella*.
**Figure S5:** Formation of hybrid BMCs and interchangeability of α‐CB and Pdu proteins in WT or Δ*pduK* strains when expressing Pdu BMCs and α‐CBs without (−) or with (+) McdAB.
**Figure S6:** Sequence similarity analysis of individual BMC‐H, BMC‐T and BMC‐P proteins between α‐CBs and Pdu BMCs.
**Figure S7:** Fluorescence imaging and SDS‐PAGE of Pdu BMCs co‐expressed with CsoS4A and CsoS4B in *Salmonella*.
**Figure S8:** SDS‐PAGE and immunoblot analysis of Pdu BMC control samples purified using the two distinct procedures.
**Figure S9:** Rubisco activity from purified α‐CBs, Pdu BMCs, and hybrid BMCs.
**Table S1:** Strains and plasmids used in this study.
**Table S2:** Primers used in this study.

**Video S1:** Zoomed‐in representation of in vivo distribution and dynamics of α‐carboxysomes (red, CbbL‐mCherry) and sfGFP‐McdA (green).

**Video S2:** Motion trajectory of α‐CBs, Pdu BMCs, and hybrid BMCs.

## Data Availability

The data that supports the findings of this study is available in Supporting Information of this article.
