# Supplementary material for: Assembly and Functional Coordination of Two Families of Metabolic Organelles in Salmonella
Source: Microb Biotechnol. 2026 Feb 3;19(2):e70301. doi: 10.1111/1751-7915.70301 (PMC12866334; doi:10.1111/1751-7915.70301)
Supplement: Supplementary file 1 — Figure S1: Fluorescence microscopy for individual α‐CB proteins in the absence of α‐CBs in S. typhimurium LT2 cells. Figure S2: Thin‐section electron microscopy (EM) of α‐CBs, Pdu BMCs and hybrid BMCs in Salmonella cells. Figure S3: Subcellular localization of α‐CBs and McdA/McdB in S. typhimurium LT2 cells. Figure S4: Assessment of McdAB, ParA, and MinD proteins on the spatial arrangement of Pdu BMCs in Salmonella. Figure S5: Formation of hybrid BMCs and interchangeability of α‐CB and Pdu proteins in WT or ΔpduK strains when expressing Pdu BMCs and α‐CBs without (−) or with (+) McdAB. Figure S6: Sequence similarity analysis of individual BMC‐H, BMC‐T and BMC‐P proteins between α‐CBs and Pdu BMCs. Figure S7: Fluorescence imaging and SDS‐PAGE of Pdu BMCs co‐expressed with CsoS4A and CsoS4B in Salmonella. Figure S8: SDS‐PAGE and immunoblot analysis of Pdu BMC control samples purified using the two distinct procedures. Figure S9: Rubisco activity from purified α‐CBs, Pdu BMCs, and hybrid BMCs. Table S1: Strains and plasmids used in this study. Table S2: Primers used in this study. [file MBT2-19-e70301-s001.docx]

**Supplementary Information**

for

**Assembly and functional coordination of two families of metabolic organelles in *Salmonella***

Ping Chang^1^, Mengru Yang^1^, Yu Chen^1^, Tianpei Li^1^, Marie Held^2^, Lu-Ning Liu^1,3*^

**This Supplementary Information file includes:**

Fig. S1 to S9

Tables S1 and S2

Video S1 and S2

Supplementary References

**
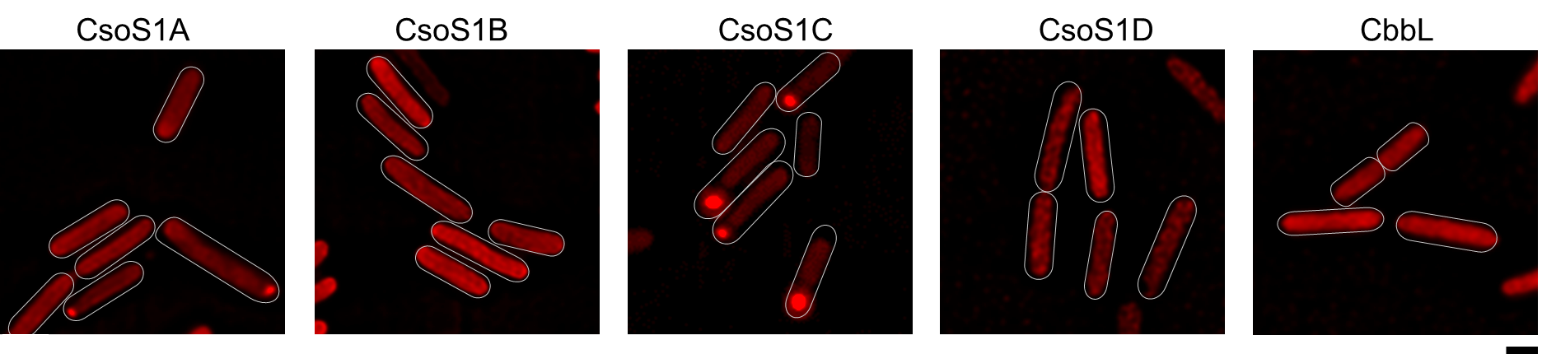
**

**Fig. S1. Fluorescence microscopy for individual α-CB proteins in the absence of α-CBs in *S.* Typhimurium LT2 cells.** *S*. Typhimurium LT2 cells expressing mCherry-tagged CsoS1A, CsoS1B, CsoS1C, CsoS1D, and CbbL were grown in microcompartment induction medium (MIM) with 0.5% succinate as the carbon source and induced with 1 mM arabinose. Scale bar: 1 μm.


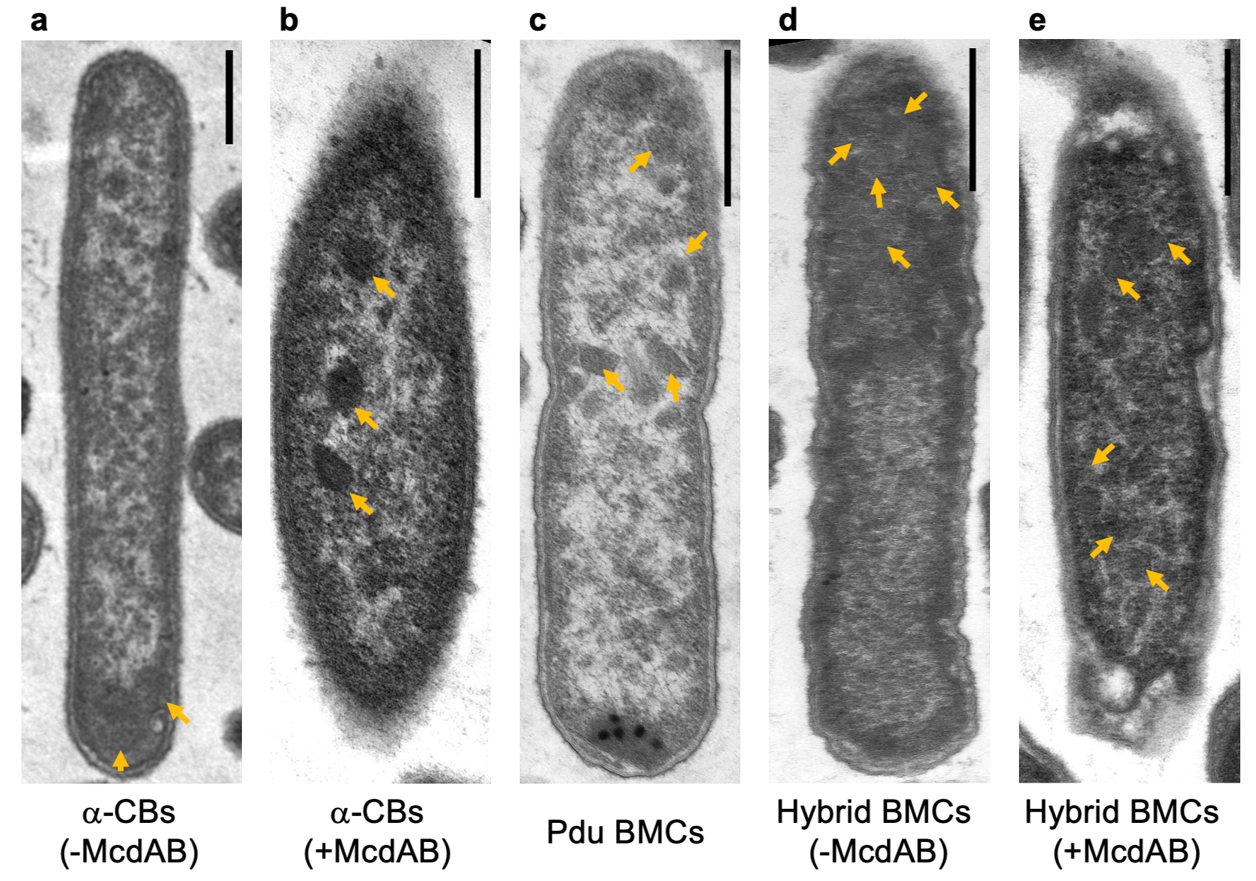


**Fig. S2. Thin-section electron microscopy (EM) of α-CBs, Pdu BMCs and hybrid BMCs in *Salmonella* cells.** α-CBs formed polar aggregates (**a**) in cells grown in MIM with 0.5% succinate in the absence of McdAB (-McdAB) compared to discrete polyhedral structures (**b**) within cells in the presence of McdAB (+McdAB). **c,** Pdu BMCs showed discrete polyhedral structures within cells grown in MIM in the presence of 1,2-PD. **d,** Hybrid BMCs without McdAB (-McdAB) showed polar aggregates when cells were grown in MIM with 1,2-PD. **e,** Hybrid BMCs in cells containing both p*Hn*CB and p*Hn*McdAB (+McdAB) grown in MIM with 1,2-PD, showed polygonal with straight edges and angular facets, typical features of BMCs. Yellow arrows indicate BMC particles discerned in the cell. Thin-section EM micrographs revealed that microcompartments were identifiable in 46% (CB), 48% (CB +McdAB), 75% (Pdu), 58% (hybrid BMCs −McdAB), and 66% (hybrid BMCs +McdAB) of cells, while the remaining cells lacked clearly visible microcompartments. The images are representative of multiple cells across independent preparations. Scale bar: 500 nm.

**
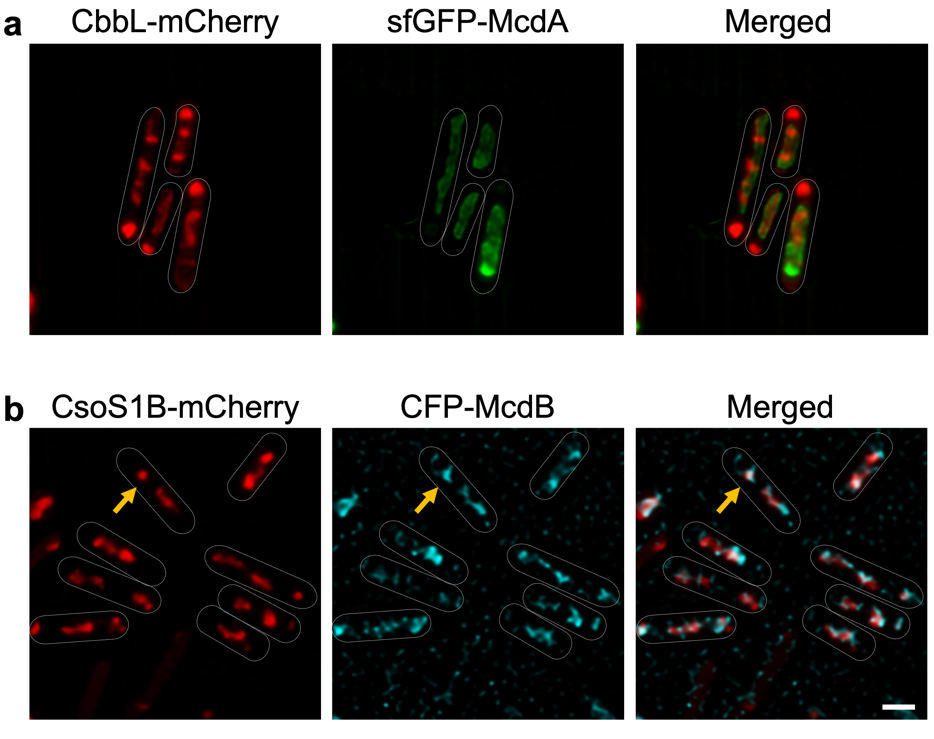
**

**Fig. S3. Subcellular localization of α-CBs and McdA/McdB in *S*. Typhimurium LT2 cells. a,** CbbL-mCherry formed discrete puncta, while sfGFP-McdA distributed throughout the cytoplasm, suggesting a possible indirect association with α-CBs. **b,** CsoS1B-mCherry and CFP-McdB colocalized as discrete patches within cells (yellow arrows), suggesting that McdB may be physically associated with α-CBs and may play a role in their positioning. Cells were grown overnight in MIM supplemented with 0.5% succinate and induced with 1 mM arabinose. Scale bar: 1 μm.

**
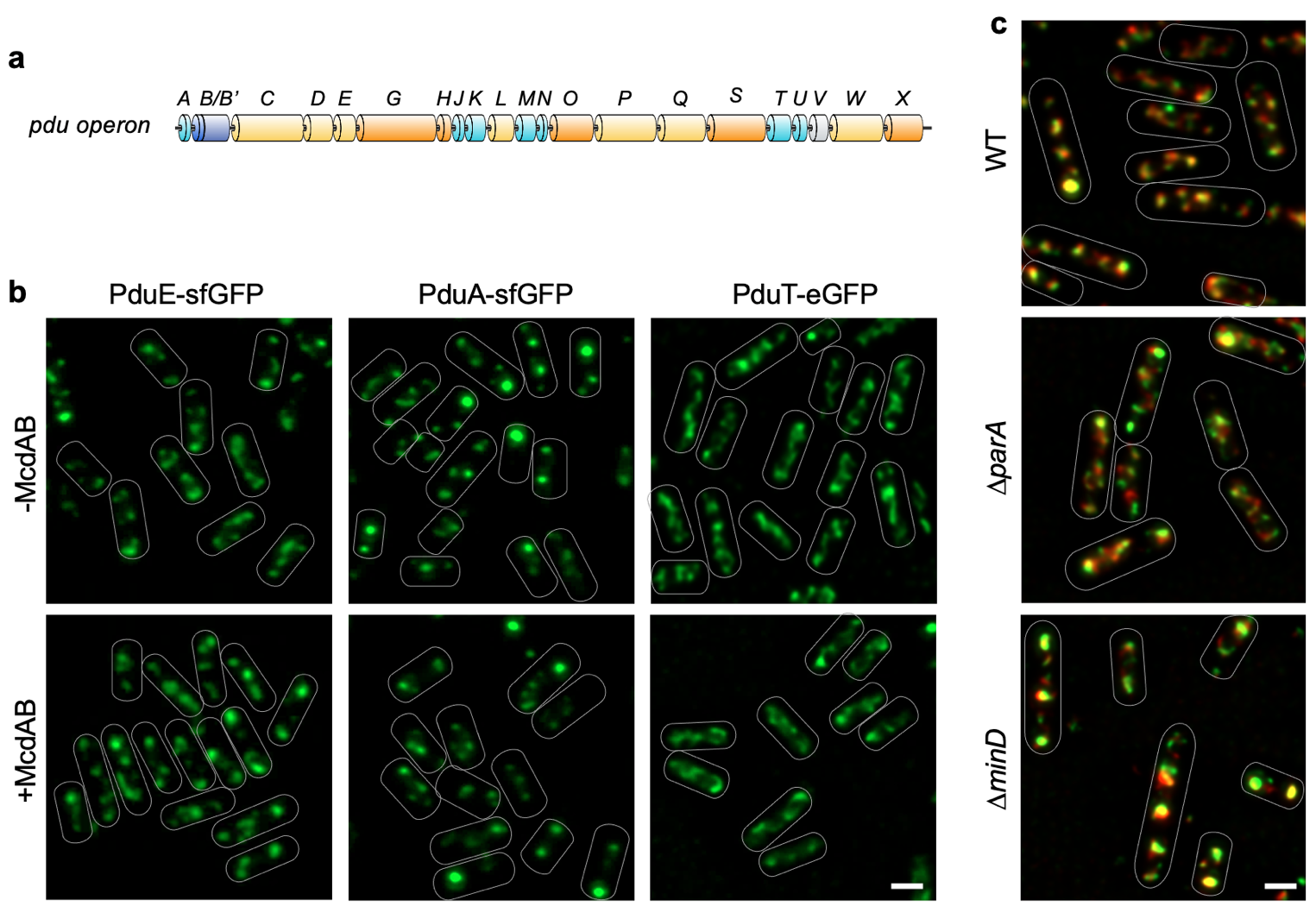
**

**Fig. S4. Assessment of McdAB, ParA, and MinD proteins on the spatial arrangement of Pdu BMCs in *Salmonella*. a,** The chromosomal *pdu* operon of *S*. Typhimurium LT2 includes structural genes (*pduABB’JKMNTU*), genes for 1,2-PD degradation (*pduCDELPQW*), vitamin B_12_ recycling (*pduGHOSX*), and *pduV*, which is proposed to be involved in filament-associated BMC movement (Yang, et al. 2022). Scale bar: 1 μm. **b,** Fluorescence imaging of LT2-*pduA-sfGFP*, LT2-*pduE-sfGFP*, and LT2-*pduT-eGFP* cells, grown in MIM supplemented with 1,2-PD, revealed typical Pdu BMC distribution patterns regardless of the presence (+) or absence (-) of McdAB, indicating that McdAB did not influence Pdu BMC positioning. **c,** The deletion of *parA* or *minD* did not alter the Pdu BMC distribution compared to discrete patches observed in WT *S.* Typhimurium LT2. Cells contained the visualized plasmid pBAD*-pduE-mCherry-PduA-sfGFP and* were cultivated in MIM supplemented with 1,2-PD. Scale bar: 1 μm.

**
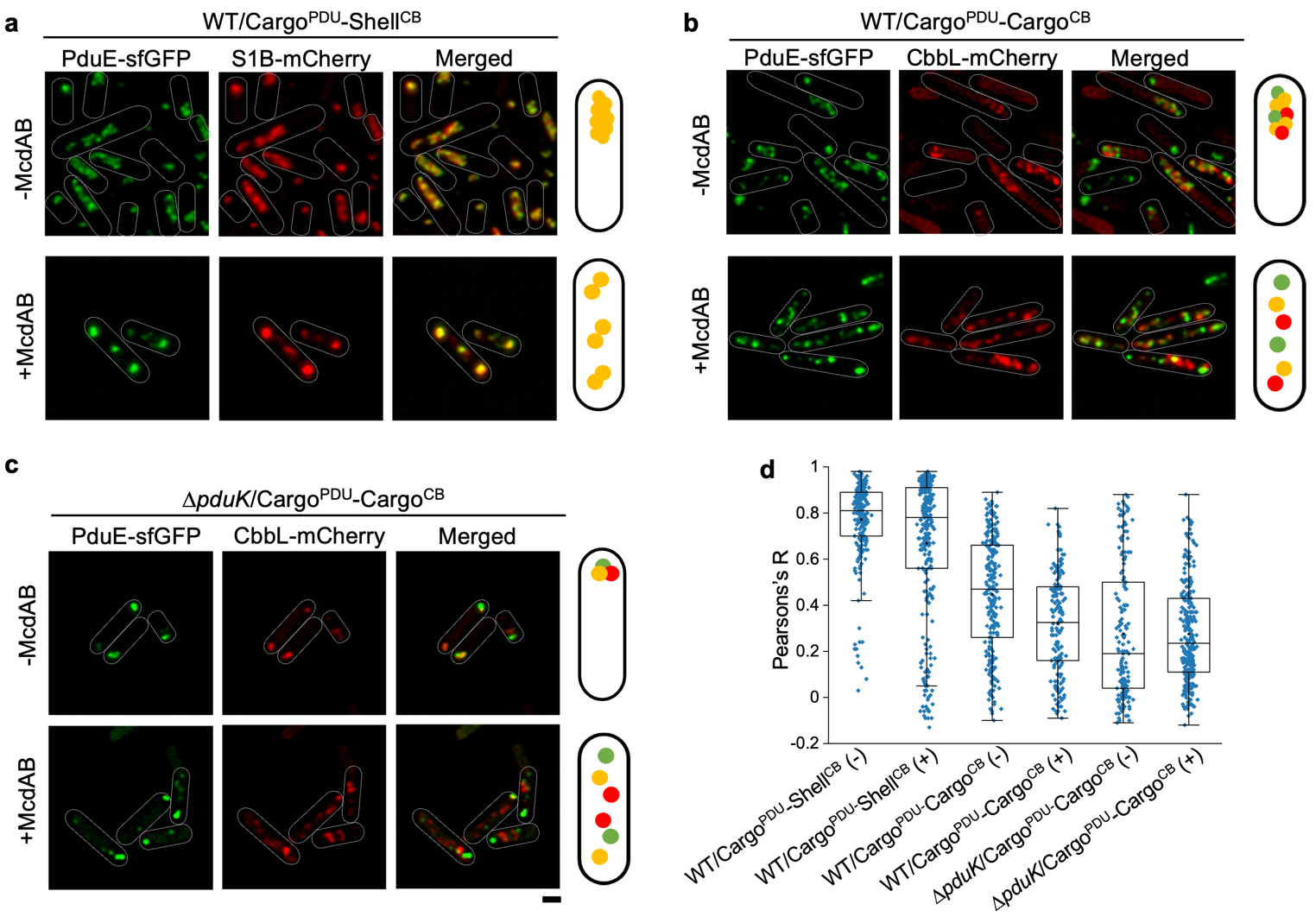
**

**Fig. S5. Formation of hybrid BMCs and interchangeability of α-CB and Pdu proteins in WT or Δ*pduK* strains when expressing Pdu BMCs and α-CBs without (-) or with (+) McdAB.** **a,** Shell and cargo proteins of α-CBs and Pdu BMCs were visualized in the WT background. **b** and **c,** Cargo proteins of α-CBs and Pdu BMCs were visualized in the WT background (**b**) or in the Δ*pduK* background (**c**). Cells were grown in MIM with 1,2-PD and induced with 1 mM arabinose. **d**, Colocalization analysis of sfGFP and mCherry fluorescence in (**a**), (**b**), and (**c**). The Pearson’s R values for all the strains are 0.76 ± 0.17 for WT/Cargo^PDU^-Shell^CB^ (-); 0.76 ± 0.26 for WT/Cargo^PDU^-Shell^CB^ (+); 0.45 ± 0.25 for WT/Cargo^PDU^-Cargo^CB^ (-); 0.32 ± 0.22 WT/Cargo^PDU^-Cargo^CB^ (+); 0.28 ± 0.29 for Δ*pduK*/Cargo^PDU^-Cargo^CB^ (-); 0.27 ± 0.22 for Δ*pduK*/Cargo^PDU^-Cargo^CB^ (+). Cargo^PDU^ represents PduE-sfGFP; Cargo^CB^ represents CbbL-mCherry; Shell^CB^ indicates CsoS1B-mCherry. n = 167 represents the number of cells. Scale bar: 1 μm.

**
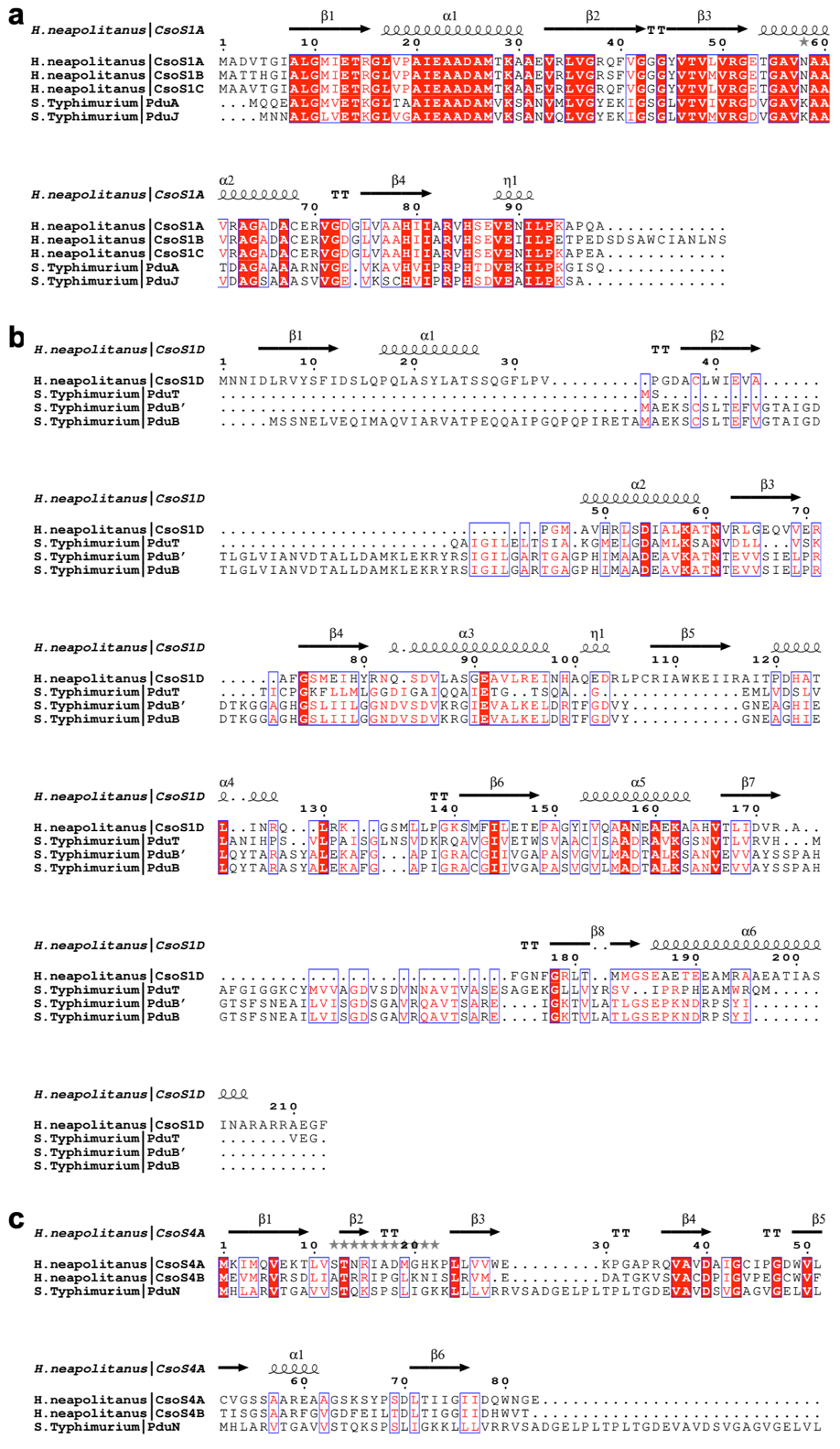
**

**Fig. S6. Sequence similarity analysis of individual BMC-H, BMC-T and BMC-P proteins between α-CBs and Pdu BMCs.** **a,** Sequence similarity analysis of BMC-H proteins: CsoS1A (Uniprot ID: P45689), CsoS1B (Uniprot ID: P45690), CsoS1C (Uniprot ID: P45688) from *H. neapolitanus,* PduA (Uniprot ID: P0A1C7) and PduJ (Uniprot ID: H9L478) from *S*. Typhimurium LT2. CsoS1A share ~ 89.8%, 97.96%, 55.32% and 57.14% identity to CsoS1B, CsoS1C, PduA and PduJ, respectively. **b,** Sequence similarity analysis of BMC-T protein CsoS1D (Uniprot ID: D0KZ73) from *H. neapolitanus,* PduB (Uniprot ID: P37449-1), PduB’ (Uniprot ID: P37449-2) and PduT (Uniprot ID: Q9XDM8) from *S*. Typhimurium LT2. CsoS1D share ~26.01%, 27.59% and 24.81% identity to PduB, PduB’ and PduT, respectively. **c,** Sequence similarity analysis of BMC-P proteins: CsoS4A (Uniprot ID: O85043), CsoS4B (Uniprot ID: O85044) from *H. neapolitanus* and PduN (Uniprot ID: Q9XDN3) from *S*. Typhimurium LT2. PduN shares ~32.93% and 28.4% identity to CsoS4A and CsoS4B, respectively.


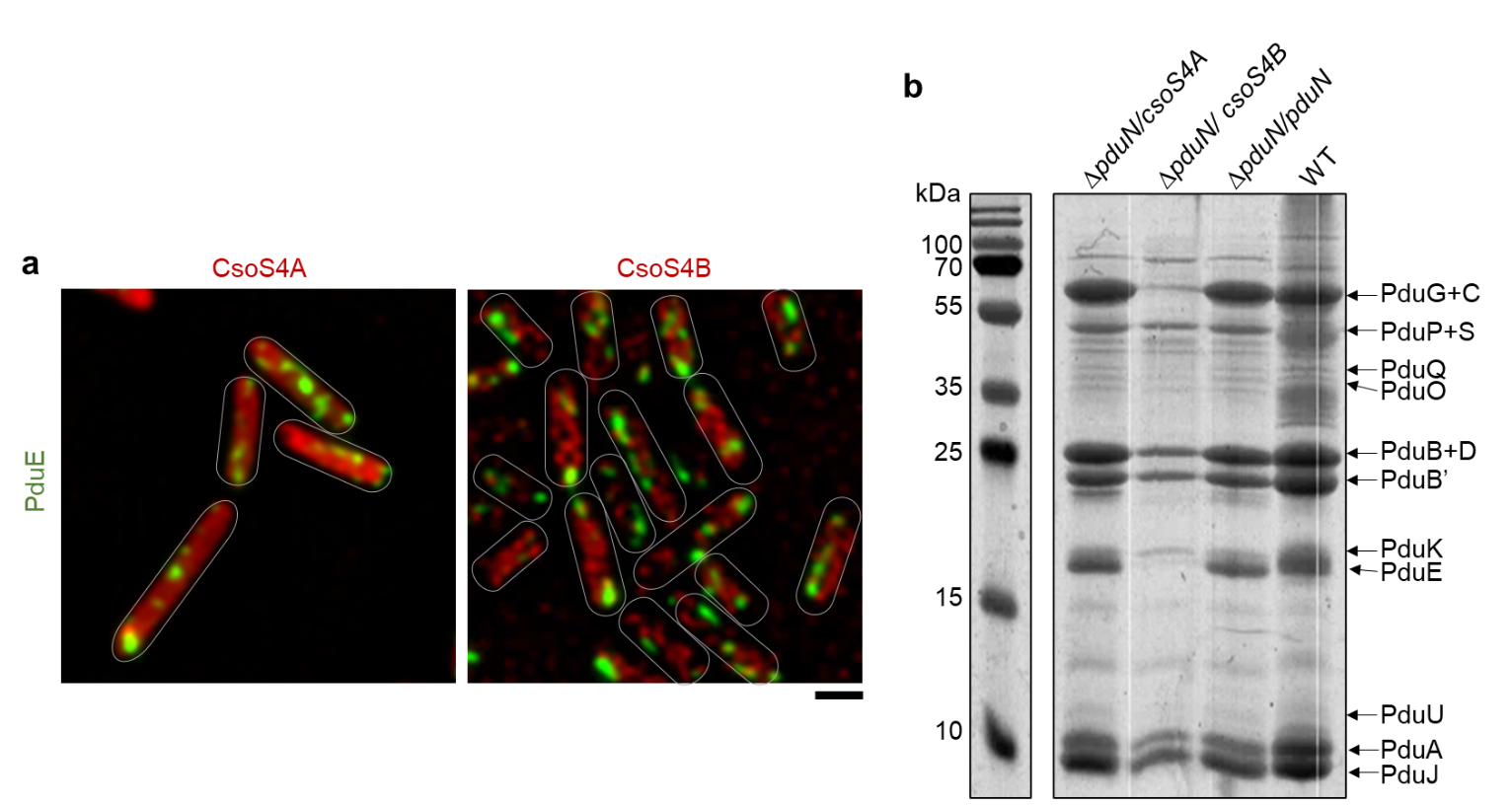


**Fig. S7. Fluorescence Imaging and SDS-PAGE of Pdu BMCs co-expressed with CsoS4A and CsoS4B in *Salmonella*.** **a,** Fluorescence images of mCherry-labeled CsoS4A and CsoS4B expressed with Pdu BMCs showed cytosol distribution. Cells were grown in MIM in the presence of 1,2-PD and were induced with 1 mM arabinose. Scale bar: 1 μm. **b,** SDS-PAGE of purified Pdu BMCs from Δ*pduN* strain expressing CsoS4A, CsoS4B or PduN. Cells were grown in MIM with 1,2-PD and 1mM IPTG as inducers.

**
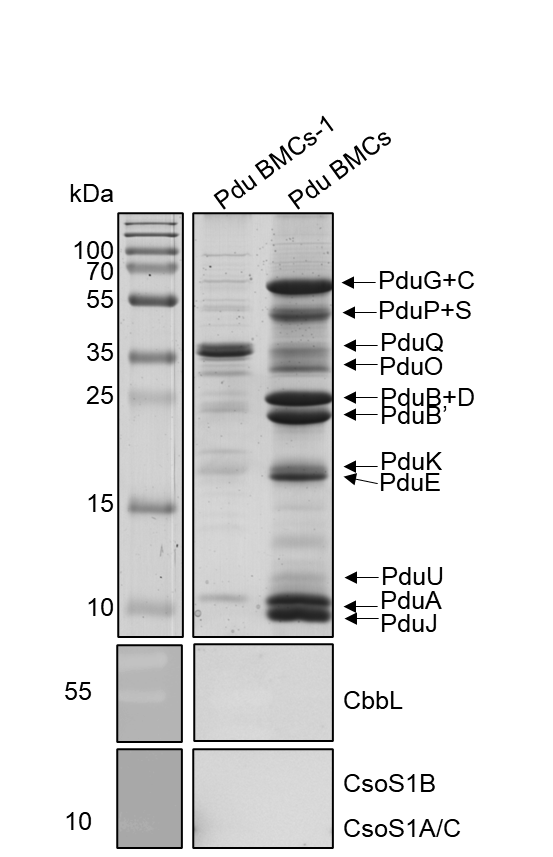
**

**Fig. S8. SDS-PAGE and immunoblot analysis of Pdu BMC control samples purified using the two distinct procedures.** Pdu BMCs-1, purified following the α-CB purification procedure, serves as a control to assess potential procedure-dependent co-purification under the α-CB purification conditions. Pdu BMCs was purified using the standard Pdu purification procedure (Yang, et al. 2020).

**
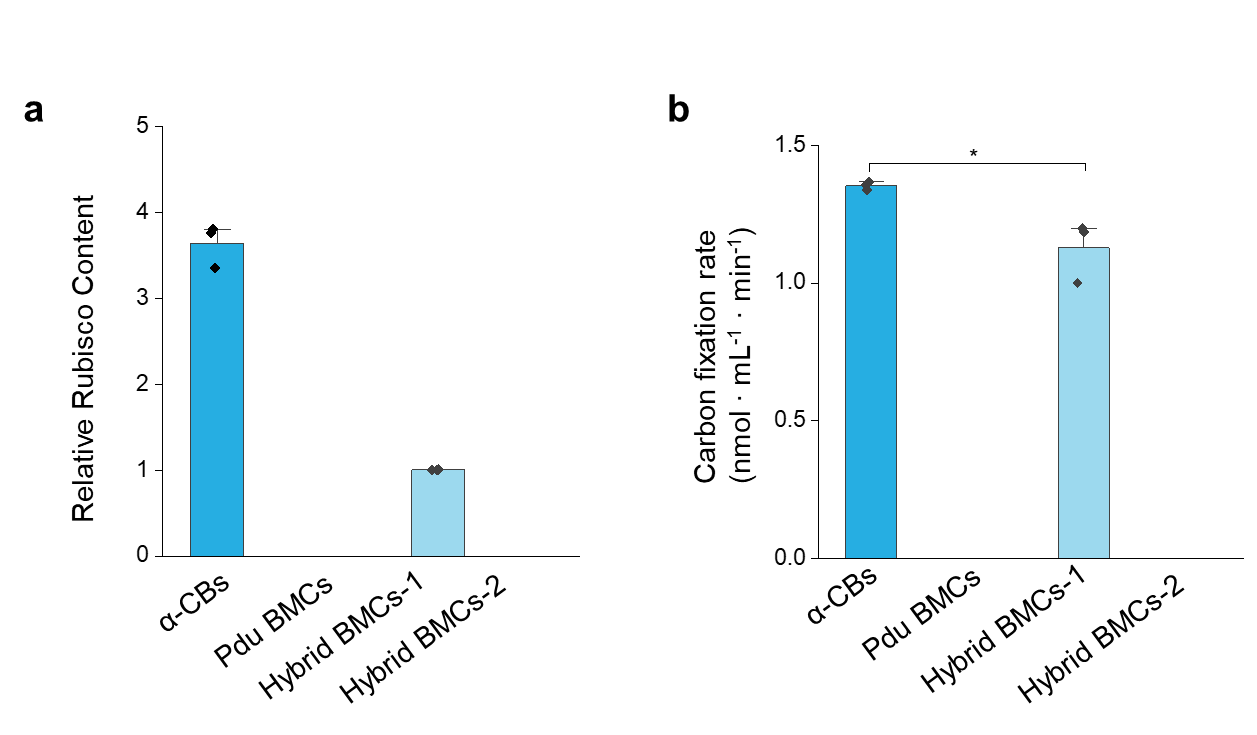
**

**Fig. S9. Rubisco activity from purified α-CBs, Pdu BMCs, and Hybrid BMCs**. **a,** Immunoblot analysis shows the Rubisco content from purified α-CBs, Pdu BMCs and hybrid BMCs samples using anti-CbbL antibody. **b,** The CO_2_ fixation rates per Rubisco were normalized by the Rubisco contents (**a**). Two-tailed unpaired *t* test, *, *p* < 0.05.

**Table S1. Strains and plasmids used in this study.**

| **Strains/plasmids** | **Description** | **Source** |
| --- | --- | --- |
| ***E. coli* derivatives:** |  |  |
| *E. coli* S17-1 λ*pir* | Cloning *E. coli* strain, *pro-82 thiE1 hsdR17 recA1 endA1* chromosome*::*RP4-2 Tc::Mu Km::Tn7*/λpir*; Tp^R^, Sm^R^ | (Simon, et al. 1983) |
|  |  |  |
| **LT2 derivatives:** |  |  |
| LT2 | Wild Type (*Salmonella enterica* serovar Typhimurium LT2) | (McClelland, et al. 2001) |
| LT2-*pduA-sfGFP* | LT2 derivative with PduA fused with sfGFP | (Yang, et al. 2022) |
| LT2-*pduE*-*sfGFP* | LT2 derivative with PduE fused with sfGFP | (Yang, et al. 2022) |
| LT2-*pduT*-*eGFP* | LT2 derivative with PduT fused with eGFP | (Yang, et al. 2020) |
| LT2-Δ*minD* | Δ*minD* | This study |
| LT2-Δ*parA*/*pBAD33tet-parA* | Δ*parA* complemented with plasmid *pBAD33tet-parA* | This study |
| LT2-Δ*pduN* | Δ*pduN* | (Yang, et al. 2022) |
|  |  |  |
| **Plasmids:** |  |  |
| pEMG | Suicide plasmid (Km^R^) | (Martinez-Garcia and de Lorenzo 2011) |
| pSW-2 | Plasmid for m-toluate-inducible expression of the I-SceI enzyme (Gm^R^) | (Martinez-Garcia and de Lorenzo 2011) |
| pBAD33-CBS1D | Complete α-carboxysome operon encoding *cbbL, cbbS* and *csoS2, csoSCA, csoS4A, csoS4B, csoS1C, csoS1A, csoS1B,* and *csoS1D* (Chl^R^) | (Sun, et al. 2022) |
| pBAD*/Myc*-His | Vector for dose-dependent expression of recombinant  Proteins (Ap^R^) | Invitrogen |
| pBAD33 | Arabinose-inducible plasmid (Chl^R^) | Addgene |
| pZ8-pTac | IPTG inducible version of pZ8-1 plasmid, to which lacIq was added, (Km^R^) | Addgene |
| pNAW55 | *frt-aph-frt-tetR-PtetA* module template plasmid, *ori*R6Kγ replicon from pEMG (Km^R^) | (Owen, et al. 2021) |
| p*Hn*CB-1 | *mCherry* cloned into p*Hn*CB vector, at the C-terminus of CbbL (Chl^R^), visualization of Cargo^CB^ | This study |
| p*Hn*CB-2 | *mCherry* cloned into p*Hn*CB vector, at the C-terminus of CsoS1B (Chl^R^), visualization of Shell^CB^ | This study |
| p*Hn*McdAB | *mcdAB* cloned into pBAD/*Myc*-His vector, His tag at the N-terminus of McdA (Ap^R^) | This study |
| p*Hn*McdAB-1 | *mcdAB* cloned into pBAD/*Myc*-His vector, sfGFP at the N-terminus of McdA and CFP at the N-terminus of McdB (Ap^R^) | This study |
| p*Hn*McdAB-2 | *mcdAB* cloned into pBAD/*Myc*-His vector, CFP at the N-terminus of McdB (Ap^R^) | This study |
| pBAD33-*csoS1A-mC* | *csoS1A* and *mCherry* cloned into pBAD33 vector (Chl^R^) | This study |
| pBAD33- *csoS1B-mC* | *csoS1B* and *mCherry* cloned into pBAD33 vector (Chl^R^) | This study |
| pBAD33-*csoS1C-mC* | *csoS1C* and *mCherry* cloned into pBAD33 vector (Chl^R^) | This study |
| pBAD33-csoS1C-mC | *csoS1D* and *mCherry* cloned into pBAD33 vector (Chl^R^) | This study |
| pBAD33-*csoS4A-mC* | *csoS4A* and *mCherry* cloned into pBAD33 vector (Chl^R^) | This study |
| pBAD33-*csoS4B-mC* | *csoS4B* and *mCherry* cloned into pBAD33 vector (Chl^R^) | This study |
| pBAD33-*csoS2-mC* | *csoS2* and *mCherry* cloned into pBAD33 vector (Chl^R^) | This study |
| pBAD33-*cbbLS-mC* | *cbbLS* and *mCherry* cloned into pBAD33 vector (Chl^R^) | This study |
| pBAD33-*csoSCA-mC* | *csoSCA* and *mCherry* cloned into pBAD33 vector (Chl^R^) | This study |
| pBAD33*tac* | Replace P*ara* with P*tac* promoter (Chl^R^) | This study |
| pBAD33*tet* | Replace P*ara* with P*tetAR* promoter (Chl^R^) | This study |
| pBAD33*tet*-*parA* | Plasmid for expression of ParA (Chl^R^) | This study |
| pBAD33*tac*-*csoS4A* | Plasmid for expression of CsoS4A (Chl^R^) | This study |
| pBAD33*tac*-*csoS4B* | Plasmid for expression of CsoS4B (Chl^R^) | This study |
| pBAD33*tac*-*pduN* | Plasmid for expression of PduN (Chl^R^) | This study |
| pBAD-*pduE-sfGFP* | *pduE:: sfGFP* cloned into pBAD/*Myc*-His (Ap^R^) | This study |
| pBAD-*EA* | *pduE::mCherry-pduA::sfGFP* cloned into pBAD/*Myc*-His at  NcoI and HindIII sites (Ap^R^) | (Yang, et al. 2022) |

Relevant antibiotic resistances are indicated by R: Ap, ampicillin; Km, kanamycin; Cm, chloramphenicol; Gm, gentamicin; Tp, trimethoprim; Sm, streptomycin. P, promoter; *ori*, origin of replication; *aph*, kanamycin resistant gene; *frt*, FLP recognition target sites.

**Table S2. Primers used in this study.**

| **Primers** | **Sequence (5’-3’)** | **Description** |
| --- | --- | --- |
| cbbl-mcherry(l1)-f | gtggacaaactcgacactcaaaatcgtggatccgctggctccgctgctggttc | Construction of p*Hn*CB-1 |
| cbbl-mcherry(l1)-r | acctcagtatgttgtgtggtacgagggactacttgtacagctcgtccatgccgcc |  |
| cbbl-mcherry(l2)-f1 | ggcggcatggacgagctgtacaagtagtccctcgtaccacacaacatactgaggt |  |
| cbbl-mcherry(l2)-r1 | ggttgctacgcctgaataagtgctgcaggcgg |  |
| cbbl-mcherry(l3)-f2 | gccgcctgcagcacttattcaggcgtagcaac |  |
| cbbl-mcherry(l3)-r2 | gccagaaccagcagcggagccagcggatccacgattttgagtgtcgagtttgtccac |  |
| s1b-mcherry(b1)-f | gcgtggtgtatcgcaaatctgaatagcggatccgctggctccgctgctggttc | Construction of p*Hn*CB-2 |
| s1b-mcherry(b1)-r | tgttgttcatgcgcatcttcccttttcactacttgtacagctcgtccatgccgcc |  |
| s1b-mcherry(b2)-f1 | ggcggcatggacgagctgtacaagtagtgaaaagggaagatgcgcatgaacaaca |  |
| s1b-mcherry(b2)-r1 | gctgtgcgtagagaaacgcacagcgcaatgacagac |  |
| s1b-mcherry(b3)-f2 | gtctgtcattgcgctgtgcgtttctctacgcacagc |  |
| s1b-mcherry(b3)-r2 | gaaccagcagcggagccagcggatccgctattcagatttgcgatacaccacgc |  |
| pbad33-pcr-f | gccaggatccgaattcgagctcgg | Construction of linearized pBAD33 |
| pbad33-pcr-r | gcccatggtatatctccttcttgaattcgc |  |
| pbad33-seq-f | atgccatagcatttttatcc | Sequencing primers |
| pbad33-seq-r | gttgctacgcctgaataagtgc |  |
| mcherry-f | ctggctccgctgctggttctggcgaattcgtgagcaagggcgaggag | Amplify mCherry with homologous sequence with flexible linker and pBAD33 |
| mcherry-r | gctcgaattcggatcctggcctacttgtacagctcgtcc |  |
| pbad33-s1a-f | caagaaggagatataccatgggcatggctgatgtaactggtattg | Construction of pBAD33-*csoS1A-mC* |
| pbad33-s1a-r | cagcagcggagccagcggatccggcttgtggcgccttag |  |
| pbad33-s1b-f | gaattcaagaaggagatataccatgggcatggcaacgactcacggtattgccctg | Construction of pBAD33-*csoS1B-mC* |
| pbad33-s1b-r | gaaccagcagcggagccagcggatccgctattcagatttgcgatacaccacgc |  |
| pbad33-s1c-f | caagaaggagatataccatgggcatggcagcagtaacaggtattg | Construction of pBAD33-*csoS1C-mC* |
| pbad33-s1c-r | gaaccagcagcggagccagcggatccagcttcaggggctttcggcaggatg |  |
| pbad33-s1d-f | caagaaggagatataccatgggcatgaacaacattgatttgcgcg | Construction of pBAD33-*csoS1D-mC* |
| pbad33-s1d-r | gcagcggagccagcggatccttagaacccttcagcgcgacgc |  |
| pbad33-s2-f | caagaaggagatataccatgggcatggggtcaaacatgcct | Construction of pBAD33-*csoS2-mC* |
| pbad33-s2-r | gcagcggagccagcggatccaccgcgcgcgccgccggag |  |
| pbad33-s4a-f | caagaaggagatataccatgggcatgaaaatcatgcaagttgag | Construction of pBAD33-*csoS4A-mC* |
| pbad33-s4a-r | accagcagcggagccagcggatccctcaccattccactgatcaat |  |
| pbad33-s4b-f | caagaaggagatataccatgggcatgcgcgttcgttccgacc | Construction of pBAD33-*csoS4B-mC* |
| pbad33-s4b-r | cagcagcggagccagcggatccagttacccagtgatcgatgatg |  |
| pbad33-cbbls-f | caagaaggagatataccatgggcatggcagttaaaaagtatagtgctgg | Construction of pBAD33-*cbbLS-mC* |
| pbad33-cbbls-r | gcagcggagccagcggatccgttgccgcggtagaccacg |  |
| pbad33-sca-f | caagaaggagatataccatgggcatgaacacccgtaacacac | Construction of pBAD33-*csoSCA-mC* |
| pbad33-sca-r | cagcagcggagccagcggatcctgcggatgcaacctcttc |  |
| pbad-pcr-f | tacgtagaacaaaaactcatctcagaagaggatctgaatagcgc | Construction of linearized pBAD |
| pbad-pcr-r | gttaattcctcctgttagcccaaaaaacgggtatggagaaacagtagagag |  |
| pbad-sfgfp-mcda-f | gggctaacaggaggaattaaccatgagcaaaggagaagaac | Construction of p*Hn*McdAB and p*Hn*McdAB-1 |
| pbad-mcda-r | tccttttgaggcggccatggttaattcctcctgttagccctcatgacgggataacctcg |  |
| pbad-cfp-f | gggctaacaggaggaattaaccatggccgcctcaaaaggagaag |  |
| pbad-mcdb-r | gatgagtttttgttctacgtatcaatctggccatacacggcgagg |  |
| pbad-hismcda-f | ggctaacaggaggaattaaccatgcatcatcatcatcatcac |  |
| pbad-ara-del-f | gataacaatttcacacaggaaacagatggtgagcaagggcgaggaggat | Construction of pBAD33*tac* |
| pbad-ara del-r | tttgcaccattcgatggtgtcttggtaacgaatcagacaattg |  |
| tac promoter-pcr-f | caattgtctgattcgttaccaagacaccatcgaatggtgcaaa |  |
| tac promoter-pcr-r | atcctcctcgcccttgctcaccatctgtttcctgtgtgaaattgttatc |  |
| tac-s4a-f | caatttcacacaggaaacagatgaaaatcatgcaagttgag | Construction of pBAD33*tac-csoS4A* |
| tac-s4a-r | ctgagatgagtttttgttctacgtattactcaccattccactgatc |  |
| tac -s4b-f | gataacaatttcacacaggaaacagatgcgcgttcgttccgacc | Construction of pBAD33*tac*-*csoS4B* |
| tac -s4b-r | ccgagctcgaattcggatcctggctcaagttacccagtgatcg |  |
| tac-pdun-f | gataacaatttcacacaggaaacagatgcatctggcacgagtcac | Construction of pBAD33*tac*-*pduN* |
| tac-pdun-r | ccgagctcgaattcggatcctggcttaacacgaaagcgtatctac |  |
| mind-f1 | agggataacagggtaatctgaattccggcttacgcattatcggcg | Construction of Δ*minD* |
| mind-r1 | aatttatcctccgaacaggcttatcctcccataaaaattccttgttaaaaaggg |  |
| mind-f2 | ccctttttaacaaggaatttttatgggaggataagcctgttcggaggataaatt |  |
| mind-r2 | cctgcaggtcgactctagaggatcccggaattgatttccggctggc |  |
| mind del-up | catgatgagaggtcgcg | Sequencing primers for Δ*minD* verification |
| mind del-down | caaggatggagatatcaccatc |  |
| para-f1 | agggataacagggtaatctgaattctgccggcagatgcagctcca | Construction of Δ*parA* |
| para-r1 | tcacatacctccattagtacgaatcacatttatcacctcatgatgtt |  |
| para-f2 | aacatcatgaggtgataaatgtgattcgtactaatggaggtatgtga |  |
| para-r2 | cctgcaggtcgactctagaggatcggataacccctccagttcagc |  |
| para del-up | cttgggcttgtccaaccc | Sequencing primers for Δ*parA* verification |
| para del-down | gttgatctcttccgttgg |  |
| para-33-f | gatagagaaaagtgaaatgcatcaccatcatcaccacgaaaatattgagcagttacgg | Construction of pBAD*33tet*-*parA* |
| para-33-r | ccgagctcgaattcggatcctggcttcacatacctccattagtacg |  |
| teta-para-f | ccgtaactgctcaatattttcgtggtgatgatggtgatgcatttcacttttctctatca |  |
| tetr-pcr-r | ttaagacccactttcacatttaag |  |

**Video S1. Zoomed-in representation of *in vivo* distribution and dynamics of α-carboxysomes (red, CbbL-mCherry) and sfGFP-McdA (green).** S. Typhimurium LT2 cells co-expressing α-CBs (p*Hn*CB-1) and McdAB (p*Hn*McdAB-1) were grown in MIM with 0.5% succinate as the carbon source and 1mM arabinose as inducer. The animation captures cells every 1 minute over a total duration of 60 minutes. The video progresses at a frame rate of 7 frames per second.

**Video S2. Motion trajectory of α-CBs, Pdu BMCs, and hybrid BMCs.** Colored lines indicate the diffusion trajectories of each BMCs, and circles represent BMC foci. All videos were taken at one frame per minute for a duration of 60 min. The video progresses at a frame rate of 7 frames per second. Scale bar, 2 µm.

**Supplementary References**

Martinez-Garcia, E., and de Lorenzo, V. 2011. “Engineering multiple genomic deletions in Gram-negative bacteria: analysis of the multi-resistant antibiotic profile of Pseudomonas putida KT2440.” *Environ Microbiol* **13**, no. 10: 2702-2716.

McClelland, M., Sanderson, K.E., Spieth, J., Clifton, S.W., Latreille, P., Courtney, L., et al. 2001. “Complete genome sequence of Salmonella enterica serovar Typhimurium LT2.” *Nature* **413**, no. 6858: 852-856.

Owen, S.V., Wenner, N., Dulberger, C.L., Rodwell, E.V., Bowers-Barnard, A., Quinones-Olvera, N., et al. 2021. “Prophages encode phage-defense systems with cognate self-immunity.” *Cell Host Microbe* **29**, no. 11: 1620-1633 e1628.

Simon, R., Priefer, U., and Pühler, A. 1983. “A Broad Host Range Mobilization System for In Vivo Genetic Engineering: Transposon Mutagenesis in Gram Negative Bacteria.” *Nat Biotech* **1**, no. 9: 784-791.

Sun, Y., Harman, V.M., Johnson, J.R., Brownridge, P.J., Chen, T., Dykes, G.F., et al. 2022. “Decoding the Absolute Stoichiometric Composition and Structural Plasticity of alpha-Carboxysomes.” *mBio* **13**, no. 2: e0362921.

Yang, M., Simpson, D.M., Wenner, N., Brownridge, P., Harman, V.M., Hinton, J.C.D., et al. 2020. “Decoding the stoichiometric composition and organisation of bacterial metabolosomes.” *Nat Commun* **11**, no. 1: 1976.

Yang, M., Wenner, N., Dykes, G.F., Li, Y., Zhu, X., Sun, Y., et al. 2022. “Biogenesis of a bacterial metabolosome for propanediol utilization.” *Nat Commun* **13**, no. 1: 2920.
